# Mechanical Investigation of CuZn30 Brass Under Conditions of Variable Axisymmetric Deformation

**DOI:** 10.3390/ma19204305

**Published:** 2026-10-13

**Authors:** Wojciech Weiler, Karol Jaśkiewicz

**Affiliations:** Metal Forming, Welding and Metrology Department, Wrocław University of Science and Technology, ul. Ignacego Łukasiewicza 5, 50-371 Wrocław, Poland

**Keywords:** cyclic torsion, monotonic torsion, strain rate, deformation path, flow stress, limit strain, CuZn30 brass

## Abstract

This paper presents the results of investigations on CuZn30 brass subjected to cyclic oscillatory torsion. The authors hypothesized that by applying specific deformation conditions under which a 180° change in the orientation of the principal components of the strain state occurs, it is possible to induce a change in the flow stress and strain. Experiments with various strain rates (0.01, 0.1 and 1 s^−1^) and different strain amplitudes (0.03, 0.06 and 0.3) were conducted at room temperature. Monotonic tests, carried out at the same temperature and under the same strain rates, served as reference results for the cyclic torsion tests. In metal forming processes, it is often assumed that elastic strains are so small compared to plastic strains that they can be neglected. However, during cyclic oscillatory torsion tests, elastic strain occurs in each deformation cycle and hinders the accurate determination of the true plastic strain. Therefore, the authors developed a dedicated method for separating elastic and plastic strains, which they applied to determine the plastic strains in individual cyclic hysteresis loops. In the cyclic torsion tests with the two smaller strain amplitudes (0.03 and 0.06), the increase in the limit strain was remarkably large—up to more than 80-fold. Such large strains were achieved at a flow stress significantly lower than that in the monotonic torsion tests, with a maximum stress drop of two and a half times. Conversely, in the tests conducted across all applied strain rates for the amplitude of 0.3, the stress was always distinctly higher, and the strain was much smaller than for the two smaller amplitudes. In all investigated cases, the results showed that for each applied strain rate, an increase in the strain amplitude leads to a very pronounced increase in the flow stress value and a reduction in the strain level, which is driven by the enhanced work-hardening of the material. The experimental results clearly demonstrate that precise control of cyclic strain parameters (strain rate and amplitude) allows for flexible tailoring of the flow stress and limit strain of the CuZn30 brass during cold forming. The authors demonstrated that forcing a 180° change in the deformation direction can lower the yield strength and, consequently, improve the deformability of the CuZn30 brass. The obtained results may contribute to the development of energy-efficient, advanced metal forming processes, as an appropriately selected cyclic deformation scheme can significantly influence both the manufacturing process and the final properties of the product.

## 1. Introduction

Scientific research on developing deformation methods that significantly reduce the magnitude of forces required to conduct plastic forming processes demonstrates that there are wide possibilities for lowering stress—and consequently reducing the work of deformation—by altering the strain path. In bulk metal forming, the influence of the strain path on the strain magnitude is more complex than in sheet metal forming, as there are far greater opportunities to control the behaviour of the deformed material. Changes in the strain path induce an evolution of the metal’s microstructure, thereby enabling a significant improvement in its deformability [1].

Improving the deformability of metals can be achieved in various ways, including structural and alloying modifications, deliberate control of the deformation history [2], thermomechanical control of process conditions, electrically assisted deformation [3], or optimization of process kinematics—for instance, by forced strain path changes or the application of complex strain paths, such as cyclic torsion [3] or torsion combined with simultaneous tension and compression [1].

The methods most commonly used to evaluate the technological deformability of metallic materials are plastometric tests, such as torsion, compression, tensile, or impact tests [4]. Plastometric testing should replicate the conditions of actual plastic forming processes as closely as possible, including complex strain paths similar to those occurring in industrial processes, such as rolling, forging, or extrusion with torsion [1].

The uniaxial tensile test is one of the simplest and, at the same time, most accurate laboratory methods for determining strain and work-hardening curves. However, a major drawback of this method is that it can only be applied to strains that are disproportionately small compared to those occurring in actual plastic forming processes [4].

The compression test is also subject to significant limitations. Specifically, it presents difficulties in eliminating friction on the working surfaces of the specimen, which leads to an inhomogeneous distribution of stress and strain [4].

On the other hand, the impact bending test does not allow for an accurate determination of the states of stress, strain, and strain rate. Therefore, this test is used only for a very rough evaluation of material deformability [4].

Against the background of the previously mentioned tests, the torsion test appears to be the most suitable for determining material characteristics at large strains and for application in investigations of forced strain path changes, such as cyclic deformation. During the torsion test, the state of stress is close to pure shear. The main advantages of this test include the capability to obtain stress–strain curves over a wide range of strains and at high strain rates; it is possible to achieve large plastic strains without prior loss of material stability—strains incomparably greater than those in compression and tensile tests. Unfortunately, a drawback of this method is the non-uniform distribution of strains, stresses, and strain rates across the cross-section of the specimen. This can hinder the interpretation of results, especially for tests conducted at elevated temperatures when phenomena causing material softening occur [1,3].

In Reference [5], the authors investigated the effect of the strain path and specimen geometry on the fatigue behavior of 304LN stainless steel, utilizing solid and thin-walled specimens subjected to tension and torsion. The material behavior during cyclic deformation, described using Masing behavior analysis and cyclic stress–strain curves, was significantly different for torsional and axial loading. The authors attributed the varying cyclic deformation responses of the steel, resulting from different strain paths, to dislocation dynamics and martensitic transformation.

In [6], tubular Ti-Cu bimetallic specimens were investigated during monotonic axial tension and axial tension combined with cyclic torsion prior to deformation at up to 1% permanent axial strain. It was found that the determined yield surface was close to the isotropic surface defined by the Huber–Mises–Hencky yield criterion. Subsequent yield surfaces were determined to evaluate the hardening and softening effects in the direction of the applied load. Only monotonic loading caused a significant increase in the tensile yield strength, whereas cyclic torsion loading led to its reduction. Microstructural studies revealed that only shear deformation affects the plastic behaviour of the bimetallic structure, as more slip systems are activated when a larger strain is applied.

In Reference [7], the authors presented the results of cyclic torsion of NiTi alloys. The described process increased the cyclic stability of elastocaloric effects by an order of magnitude, extending the cyclic lifetime of coarse-grained NiTi shape memory alloys from 285 to 1114 cycles, while maintaining a stable, adiabatic temperature drop of approximately 8 K. The authors attributed this hardening to the gradient structure, which significantly suppresses dislocation generation during the subsequent cyclic phase transformation.

In References [8,9], the effect of cyclic torsion on the microstructure of a single-phase Al_0.1_CoCrFeNi multi-component alloy was investigated. In [8], the authors studied a fine-grained material subjected to varying cumulative plastic strains ranging from 0.1 to 14.6. The results showed that conventional single-slip dislocations dominate plastic deformation at the initial stage of straining. At a strain of up to 1.2, a large number of dislocation locks, formed by dislocation interactions, induce extensive proliferation of multi-slip dislocations within the grain interiors. At strains above 4, a large number of individual dislocations are gradually organized into massive, two-dimensional, multi-slip dislocation segments. At strains above 8, finer but numerous, three-dimensional low-angle dislocation cells are formed. The characteristic features of the hierarchical dislocation cell structure at the specimen level in metals with low stacking fault energy are mainly caused by the gradient distribution of small but highly cumulative plastic strains, which are closely related to the enhanced activity of multiple dislocation slips. Conversely, in [9], the mechanism of intrinsic dislocation activity occurring during cyclic torsion was investigated in the same Al_0.1_CoCrFeNi alloy, but with a coarse-grained microstructure, i.e., with one-millimetre-sized grains. In contrast to the extensive dislocation proliferation and multiple slips in the fine-grained alloy, in this coarse-grained material, fewer dislocation slips and parallelly aligned locks are preferentially activated, which consequently promotes the generation of numerous geometrically necessary boundaries (GNBs) within the large grain interiors. With a further increase in strain, the density of GNBs gradually increases and the spacing between them decreases, which also accelerates the formation of an ultra-fine dislocation cell pattern.

In References [10,11,12,13,14], the authors analysed torsion tests of magnesium alloys. In Reference [10], the relationship between strain and grain size of a magnesium alloy subjected to cyclic tension–torsion loading was investigated by conducting experiments with different strain paths and analysing the microstructure. The experimental results showed that the stress–strain curves exhibit different characteristics depending on the deformation mechanism. Microstructural analysis revealed that the statistical correlation between twin thickness and grain size is not obvious, but the number of twin nuclei remains stable and is related to the grain boundary area, and with an increase in strain amplitude, additional twin nuclei are formed within the grain area. The authors of Reference [11] conducted a detailed review of the complex mechanical properties of magnesium alloys subjected to cyclic deformation. Material fatigue is influenced by, among other things, dislocation slip and twinning. Reference [12] is also a review paper that summarizes the fundamentals of mechanics, microstructure evolution, mechanical property improvement, and numerical simulation of magnesium alloys subjected to torsion. In [13], the authors investigated the feasibility of improving the mechanical properties and corrosion resistance of a Mg-3Zn-0.2Ca alloy through pre-torsion treatment and determining the optimal shear strain. The specimens were subjected to pre-deformation by twisting at various angles. The effect of torsion on the studied material was characterized by microstructural analysis, mechanical testing, and corrosion testing. It was shown that the pre-torsion treatment can refine the grain size and induce twinning. The surface hardness increases with the twist angle. The yield strength and tensile strength initially increase and then decrease with the increase in twist angle, while the ductility decreases with the twist angle. The authors concluded that pre-torsion treatment can effectively modify the microstructure of the Mg-3Zn-0.2Ca alloy, improving both its mechanical properties and corrosion resistance, which makes it more suitable for biomedical implant applications. The authors of Reference [14] proposed novel, coupled pre-treatment methods involving high-angle pre-torsion and low-temperature aging to improve the fatigue resistance of a rolled ZK60 magnesium alloy. Microstructural studies revealed that pre-torsion significantly enhanced twin formation and increased dislocation density. Mechanical testing showed that the combined pre-treatment yielded a better combination of strength and ductility. The results suggest that the combined pre-torsion and low-temperature aging treatments can lead to improved mechanical properties and fatigue resistance of the ZK60 magnesium alloy.

In [15], investigations of RG7 tin–zinc–lead bronze under conditions of cyclic bending, cyclic torsion, and combined cyclic bending and torsion were presented. Based on microscopic observations, it was found that the fatigue cracks were similar across all deformation conditions, indicating that the qualitative nature of the fatigue process did not differ in these cases.

In Reference [16], the authors conducted cyclic torsion tests on copper wires with and without surface passivation. A typical work-hardening effect was observed during symmetrical cycles. A greater increase in strength occurred during the torsion of the passivated copper wires.

Based on the above review of recent papers, it appears that—compared to the number of studies presented on magnesium alloys, for instance—relatively few torsion tests on copper alloys have been conducted recently. Therefore, the authors of this manuscript undertook the investigation of cyclic torsion of CuZn30 brass.

This brass is primarily used for the manufacturing of thin-walled products, such as cartridge cases, radiator tubes, and heat exchangers tubes. This application stems directly from the fact that CuZn30 exhibits the highest plasticity (deep drawing capability) among all binary brasses. This allows for the metal forming of products with complex geometries without material cracking. While conventional manufacturing methods for the aforementioned products are well-established, the drive to reduce the number of manufacturing stages in the technological process necessitates the application of innovative methods, including the use of forced strain path changes. In this context, research on forming processes under an additional oscillatory load represents a distinct research gap. The selection of CuZn30 brass for this study is justified by its industrial applications and its low stacking fault energy (SFE). Most alloys subjected to strain path change investigations (such as pure copper, aluminium, or steels) possess medium to high SFE. Consequently, the microscopic mechanism leading to the stress drop in CuZn30 differs from that in other studied metals. Demonstrating that cyclic torsion increases the formability of this specific alloy without causing premature cracking has direct implications for the design of modern metal forming processes.

The authors present an analysis of the effect of the forced strain path, achieved during minor-cyclic torsion of CuZn30 brass, on stress and strain. The term “minor-cyclic deformation” is explained in detail later in the manuscript (Section 2.3)—it refers to a cyclic deformation process characterized by large plastic strains in individual cycles, where material failure occurs after a number of cycles not exceeding 10^4^.

## 2. Experimental Equipment and Materials

### 2.1. Experimental Equipment

**Figure 1:**
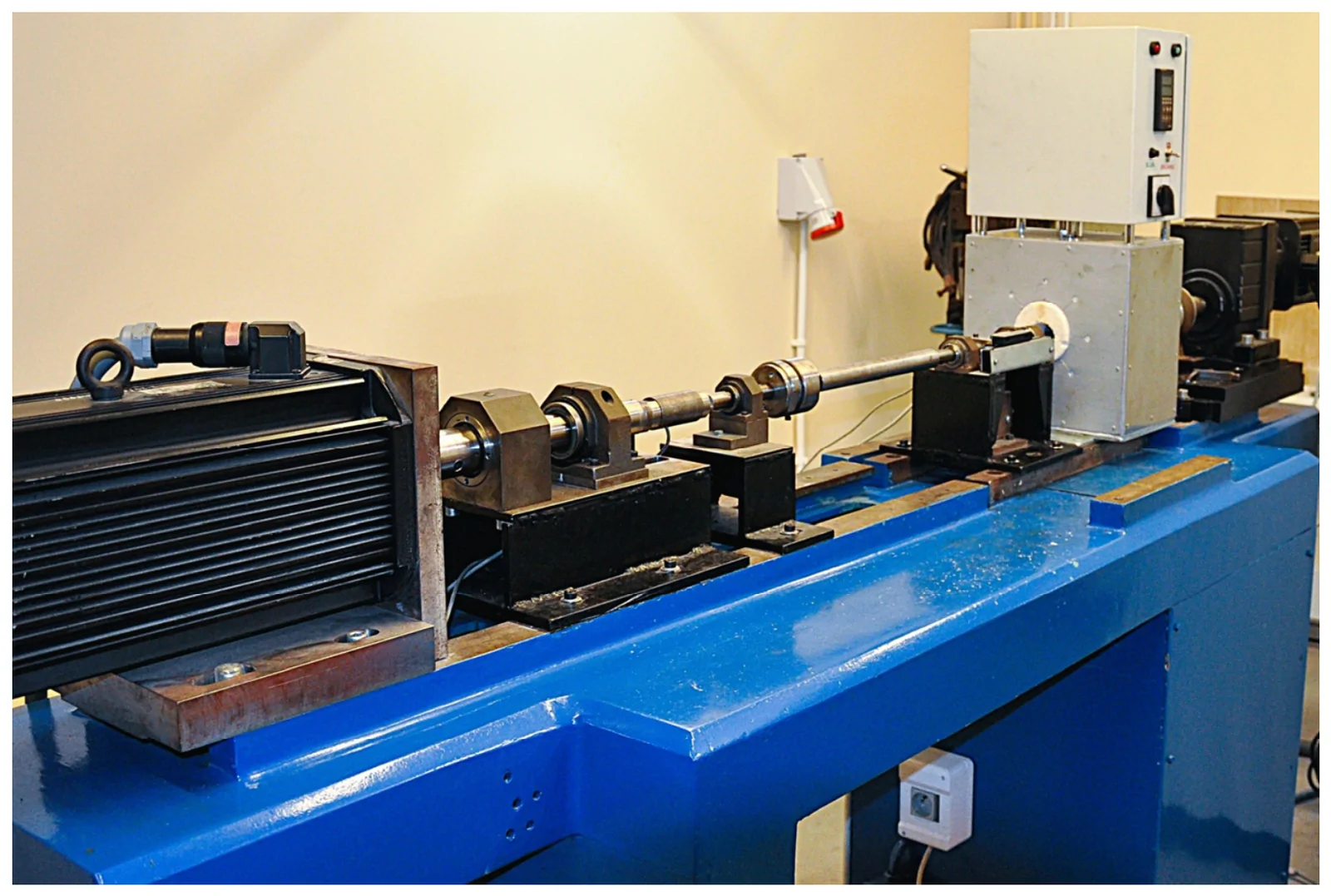
Plastometer used for torsion tests.

The investigations were conducted on a custom-designed and custom-built test rig, shown in Figure 1. The patented proprietary plastometer enables the realization of complex strain paths. It allows for testing over a wide range of strain amplitudes, at temperatures from room temperature up to 1200 °C, at strain rates ranging from 10^−3^ to 10^2^ s^−1^. The plastometer is equipped with two Lenze (Aerzen, Germany) asynchronous electric motors—model MDFKARS100-22 with a power output of 13.2 kW and a torque of 36 Nm, and model MDFKARS112-22 with a power output of 11 kW and a torque of 61.4 Nm. Due to the application of these inverter-controlled motors, which feature an extremely short start-up time, very high and stable strain rates can be achieved. The rotational speed stability of each motor in the range from 0.5 to 1000 RPM is ±0.1%, while the speed rise time from 0 to 1000 RPM does not exceed 10 ms.

For torque measurement, sensors equipped with high-precision TF-3/120 foil electrical resistance strain gauges (Tenmex, Łódź, Poland), along with a compensating strain gauge system, were utilized. To achieve higher measurement sensitivity, three interchangeable sensors with torque measuring ranges of 0–3 Nm, 0–10 Nm, and 0–40 Nm were employed. The rotational speed and angular displacement are measured using the motor servodrive. For axial force measurement, a sleeve-type sensor with bonded strain gauges was used. To obtain higher measurement sensitivity, three interchangeable sleeve sensors with measuring ranges of 0–3 kN, 0–7 kN, and 0–20 kN were applied.

In addition to monotonic and cyclic torsion, the presented plastometer also enables the execution of the following tests:

- Tension;
- Compression;
- Arbitrary torsion combined with tension or compression, including deformation holding times.

### 2.2. Tested Material

The CuZn30 brass intended for this study was cast as ingots from pure copper and zinc, which significantly reduced additional impurities commonly found in commercial (industrial) alloys. The presence of such impurities could hinder the correct interpretation of phenomena occurring in the material during deformation in plastometric tests. The ingots were extruded into round bars with a length of approximately 2500 mm and a diameter of 20 mm. To minimize discrepancies associated with varying conditions during the metallurgical process, the material was produced from a single heat to ensure an identical chemical composition across all specimens. The chemical composition of the brass was determined using a GDS500A Glow Discharge Spectrometer, Leco, St. Joseph, MI, USA and is presented in Table 1.

The supplied bars were cut into sections approximately 500 mm in length to fit into the furnace and subjected to annealing to homogenize the microstructure (eliminating elongated grains, banding, and deformation texture). The homogenization annealing was performed at 700 °C for 60 min. For CuZn30 brass, there are no strict or rigorous heating rate limitations because—unlike steel—no allotropic transformations occur, meaning the material does not undergo a crystal structure change with temperature that would necessitate slow heating. CuZn30 heats relatively quickly and uniformly throughout its volume. The target temperature of 700 °C was reached by placing the material directly into a resistance furnace preheated to the operating temperature, resulting in a rapid temperature rise. A bar with a diameter of 20 mm assumes the furnace temperature uniformly and very rapidly without the risk of thermal cracking or internal stresses. Following annealing, the bars were water-quenched to prevent further grain growth and retain the recrystallized microstructure.

**Figure 2:**
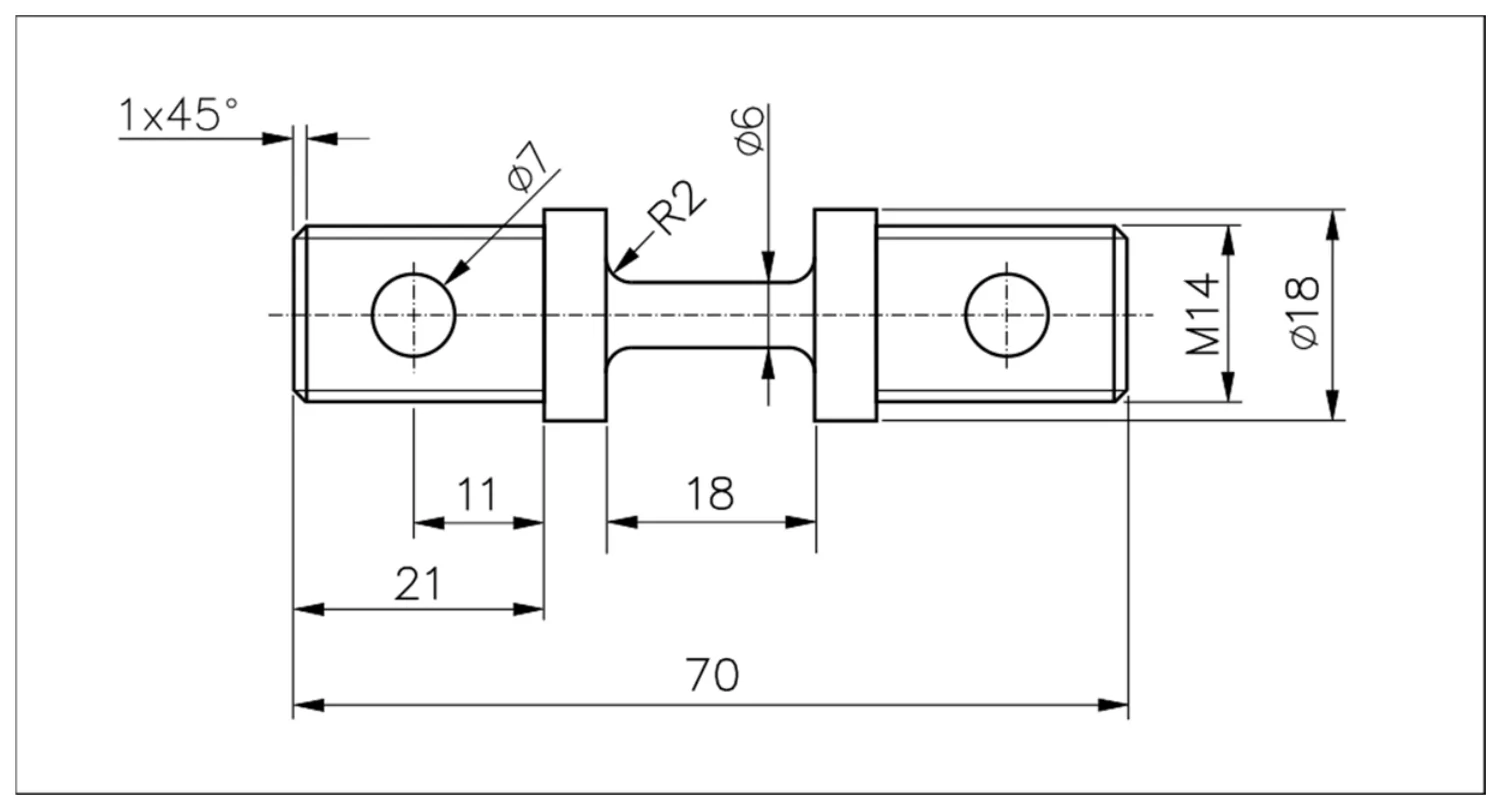
Shape and dimensions of the tested specimens.

Specimens with the geometry and dimensions shown in Figure 2 were machined from the heat-treated material. The brass bars were turned on a TUR 50 × 1500 universal lathe (Fabryka Automatów Tokarskich PONAR-Wrocław, Wrocław, Poland). For the cutting process, a ground, single-sided, rhombic DCGT 11T304-AL H01 turning insert (Korloy Europe GmbH, Oberursel, Germany) was used. The insert featured a positive geometry and a 55° included angle, designed for precision finishing and medium-roughing of high-ductility, long-chipping materials such as aluminium or copper alloys.

During rough machining (transitioning from a diameter of 20 mm to 12 mm), the spindle speed was 1600 rpm, corresponding to a cutting speed of 100 m/min. During finish turning, i.e., transitioning from a diameter of 12 mm to the target diameter of 6 mm (+0.05/0), the spindle speed was increased to 2000 rpm, corresponding to a cutting speed of 75 m/min. The feed rate during rough turning was 0.13 mm/rev, and 0.08 mm/rev during finish turning, while manual feed was applied when machining the transition fillet (between the gauge section and the gripping section of the specimen). The depth of cut for the gauge section of the specimen was gradually reduced from roughing to finishing: from 1 mm, through 0.7 mm, down to 0.4 mm. A lower depth of cut was not applied because CuZn30 brass is highly susceptible to work hardening. Below a depth of cut equal to the tool nose radius, the insert ceases to cut with its main cutting edge. This alters the force distribution unfavourably, where the thrust force component starts to dominate while the feed force component decreases. Such machining conditions promote the plowing effect, leading to severe work hardening of the material. This is particularly critical for materials prone to strain hardening, as is the case for the investigated brass. During turning, the specimen was cooled using Blasocut 2000 Universal water-soluble cooling and lubricating emulsion (Blaser Swisslube AG, Hasle-Rüegsau, Switzerland).

Prior to the plastometric testing, the machined specimens were subjected to stress-relief annealing at 700 °C for 10 min to eliminate residual stresses induced by turning. This was necessary because the quality of the machined surface and the condition of the surface layer significantly affect the mechanical properties. In strain-hardened materials—such as CuZn30 brass, among others—deformation of the surface layer leads to changes in the residual stress values. During turning, tensile stresses are generated in the surface layer of the workpiece in front of the cutting tool edge, whereas compressive stresses are induced after the lathe tool pass. The target specimen temperature of 700 °C was reached by placing them directly into a furnace preheated to the operating temperature, resulting in a rapid heating rate. Following this brief annealing, the specimens were water-quenched.

Since the stress-relief annealing was conducted in a furnace without a protective atmosphere, the gauge section of each specimen had to be cleaned using P600 grit sandpaper to remove the thin oxidized surface layer. The machined specimen, clamped in the lathe, was rotated at a spindle speed of 350 rpm (corresponding to a cutting speed of just under 7 m/min), while a narrow strip of sandpaper was applied manually. Due to these process parameters—specifically the cutting speed and the nature of the operation—this procedure can hardly be classified as true grinding; hence, the authors prefer the term “surface cleaning with sandpaper”.

The specimens prepared in this manner were then deformed in the plastometric torsion tests.

### 2.3. Experimental Methodology

The material response to a forced strain path change is closely related to the angle between the displacement velocity vectors in the subsequent deformation stages. For this angle to affect the dislocation structure, it must be sufficiently large, specifically at least 90° [3].

If the angle between the displacement velocity vectors in subsequent deformation stages changes by approximately 180°, we are dealing with reverse deformation, i.e., cyclic deformation—the most commonly used method of complex deformation. It is widely known that different types of cyclic deformation are distinguished depending on the number of cycles required for material failure. In high-cyclic deformation, material failure occurs after no fewer than 10^5^ cycles. When the material ruptures after 10^4^ to 10^5^ cycles, we refer to it as low-cyclic deformation. However, these two types of cyclic deformation concern fatigue strength rather than metal forming processes. Therefore, years ago, researchers from the Department of Metal Forming, Welding and Metrology at Wrocław University of Science and Technology introduced a new term: “minor-cyclic deformation”. This term is used to define a deformation process in which significant plastic strains occur in subsequent cycles, and material fracture can take place after just a few deformation cycles [1,3]. The process of minor-cyclic deformation must be clearly distinguished from that of low-cyclic. Deformation carried out in this manner leads to a cyclic change in slip systems. If the deformation is large, the effect of heterogeneous plastic flow can be neglected. If it is small, elastic strains play a significant role. A strain value equal to 1 was established as the boundary between large and small strains [3].

The cycle asymmetry ratio is described by the formula:

(1) $$S_{c}=\frac{\varepsilon_{a1}}{\varepsilon_{a2}},$$

where:

$\varepsilon_{a1}$—total strain amplitude in the primary direction;

$\varepsilon_{a2}$—total strain amplitude in the reverse direction (opposite to the primary one).

**Figure 3:**
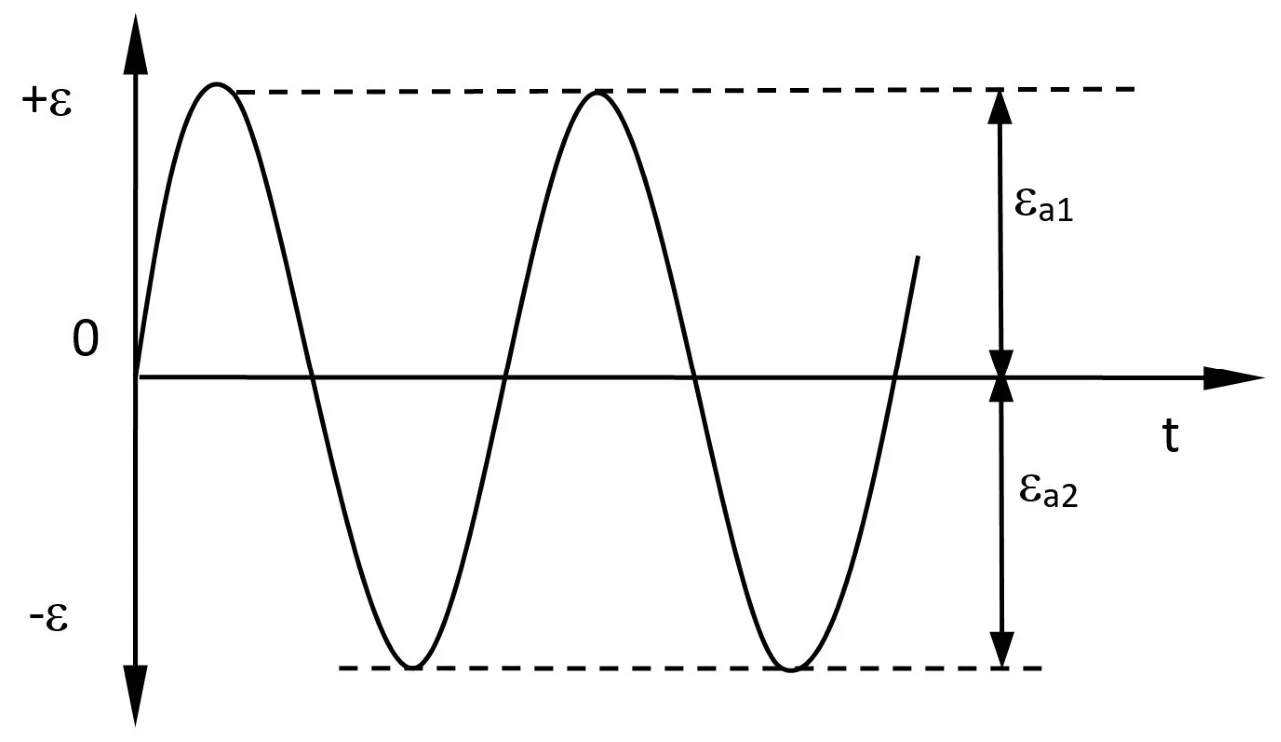
Symmetrical cyclic deformation vs. time [3].

In this study, cyclic symmetrical deformation was applied, where the *S_c_* ratio is equal to 1 (Figure 3).

In the torsion test, the torque is determined as a function of the specimen’s twist angle [17,18], and these quantities are subsequently converted into equivalent stress and equivalent strain [19,20]. In this work, the classical method was applied, in which the flow stress derived from the torque is described by the equation:

(2) $$\sigma_{p}=\sqrt{3}\tau =\sqrt{3}\frac{3M(m+n)}{2\pi_{rz}^{3}},$$

and the equivalent strain, in turn, is described by the equation:

(3) $$\varepsilon_{red}=\frac{1}{\sqrt{3}}\gamma =\sqrt{3}\frac{r_{rz}\omega }{l_{rz}},$$

where:

*M*—torsional moment;

*n*—strain hardening exponent;

*m*—strain rate sensitivity exponent;

*ω*—specimen torsional angle;

*r_rz_*—specimen true radius;

*l_rz_*—specimen true length;

*γ*—non-dilatational strain.

The strain hardening factor *n* is defined by the following equation [17,18]:

(4) $$n=\frac{N}{M}\frac{\delta M}{\delta N},$$

and the strain rate sensitivity factor *m* is defined by the following equation [17,18]:

(5) $$m=\frac{\dot{N}}{M}\frac{\delta M}{\delta \dot{N}},$$

where:

*M*—torsional moment;

*N*—number of specimen rotations;

$\dot{N}$—specimen torsion rate.

The strain amplitude was determined from Equation (3) based on the specimen’s twist angle; for example, for a specimen twist angle of *ω =* 180°, the strain amplitude was *ε_ac_* = 0.3.

In most analyses concerning monotonic plastic deformation processes, it is typically assumed that elastic strains are negligibly small compared to plastic strains. In contrast, in cyclic deformation processes, elastic strains occur in every cycle, and their cumulative sum can be highly significant; therefore, they can no longer be neglected. Knowledge of the elastic strains in subsequent strain hysteresis loops is essential for determining the true stress–strain curves, as during oscillatory torsion tests, elastic strain occurs—as previously indicated—during each deformation cycle and hinders the correct reading of plastic strain.

**Figure 4:**
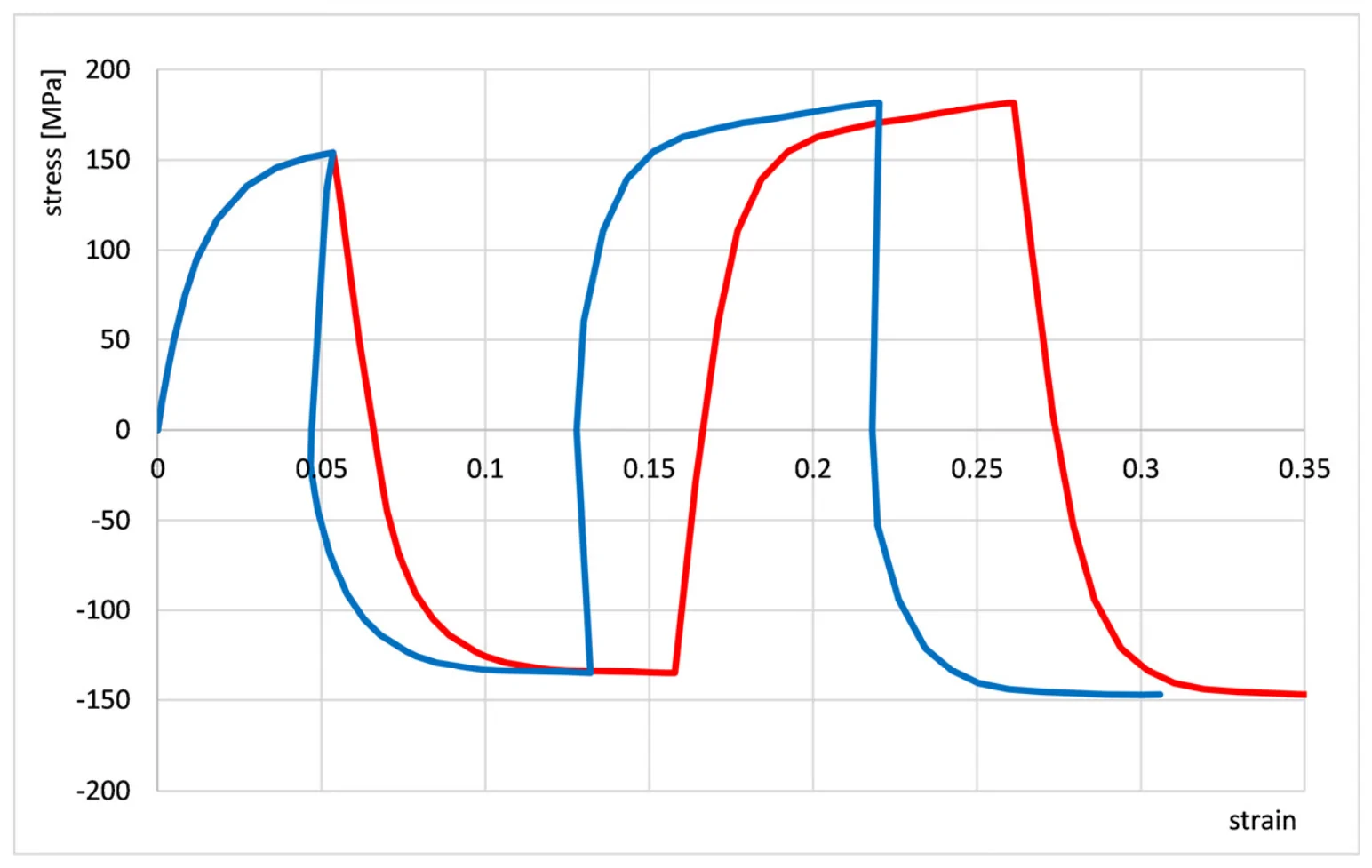
Comparison of the cyclic oscillatory torsion curve at a strain amplitude A = 0.06 containing elastic strains (red line) with the true curve (corrected, blue line) [3].

Figure 4 compares the stress curves for cyclic oscillatory torsion at a strain amplitude of 0.06 as a function of total strain (plastic strain + elastic strain, indicated in red) and as a function of plastic strain only (indicated in blue). The comparison indicates that even at a low total strain—for instance, 0.27—the contribution of elastic strains for the given specimen reaches approximately 20%, which leads to significant errors in the interpretation of the obtained results. With further deformation, this contribution increases. Therefore, the authors developed a method to separate the elastic and plastic strains. The calculations of plastic strains in individual cyclic strain hysteresis loops were performed using a program within the plastometer software. Consequently, in the stress–strain curves presented in this manuscript, only plastic strains are analysed, as the elastic strains have been eliminated from the total strains. The elimination of elastic strains results in a very pronounced shortening of the curves.

In the torsion test, changes in specimen dimensions depend on the deformation conditions. During torsion at room temperature, a slight elongation of the specimen occurs, which is associated with texture development. On the other hand, deformation at elevated temperatures leads to a minor shortening of the specimen caused by dynamic recrystallization, nucleation, and selective grain growth. However, these changes are so small that they were neglected in the stress and strain calculations. In this work, it was also assumed that the radius and length of the specimen do not change during deformation.

Prior studies on various methods of determining flow stress curves as a function of strain [1,3] have shown that using strain rates higher than $\dot{\varepsilon }$ = 1 s^−1^ causes a significant temperature increase in the deformed specimen. This is due to the conversion of plastic deformation work into heat. Under such deformation conditions, a highly non-uniform temperature distribution was obtained across the cross-section and along the length of the specimen, resulting from intensive heat dissipation through the plastometer grips in which the specimen was clamped. This distribution results in the softening of the specimen in its central section. At lower strain rates, the rate of heat generation is much lower, and the torsion process can then be assumed to occur under near-isothermal conditions. Therefore, the strain rates used in this study were limited to those that do not cause a significant temperature rise in the deformed specimen.

For the investigation of CuZn30 brass at room temperature, the following deformation variants were selected:

- Monotonic (unidirectional) torsion at a strain rate of $\dot{\varepsilon }$ = 0.01 s^−1^;
- Monotonic (unidirectional) torsion at a strain rate of $\dot{\varepsilon }$ = 0.1 s^−1^;
- Monotonic (unidirectional) torsion at a strain rate of $\dot{\varepsilon }$ = 1 s^−1^;
- Symmetrical (fully reversed) cyclic torsion at a strain rate of $\dot{\varepsilon }$ = 0.01 s^−1^ and a strain amplitude of *ε_ac_* = 0.03;
- Symmetrical (fully reversed) cyclic torsion at a strain rate of $\dot{\varepsilon }$ = 0.01 s^−1^ and a strain amplitude of *ε_ac_* = 0.06;
- Symmetrical (fully reversed) cyclic torsion at a strain rate of $\dot{\varepsilon }$ = 0.01 s^−1^ and a strain amplitude of *ε_ac_* = 0.3;
- Symmetrical (fully reversed) cyclic torsion at a strain rate of $\dot{\varepsilon }$ = 0.1 s^−1^ and a strain amplitude of *ε_ac_* = 0.03;
- Symmetrical (fully reversed) cyclic torsion at a strain rate of $\dot{\varepsilon }$ = 0.1 s^−1^ and a strain amplitude of *ε_ac_* = 0.06;
- Symmetrical (fully reversed) cyclic torsion at a strain rate of $\dot{\varepsilon }$ = 0.1 s^−1^ and a strain amplitude of *ε_ac_* = 0.3;
- Symmetrical (fully reversed) cyclic torsion at a strain rate of $\dot{\varepsilon }$ = 1 s^−1^ and a strain amplitude of *ε_ac_* = 0.03;
- Symmetrical (fully reversed) cyclic torsion at a strain rate of $\dot{\varepsilon }$ = 1 s^−1^ and a strain amplitude of *ε_ac_* = 0.06;
- Symmetrical (fully reversed) cyclic torsion at a strain rate of $\dot{\varepsilon }$ = 1 s^−1^ and a strain amplitude of *ε_ac_* = 0.3.

**Figure 5:**
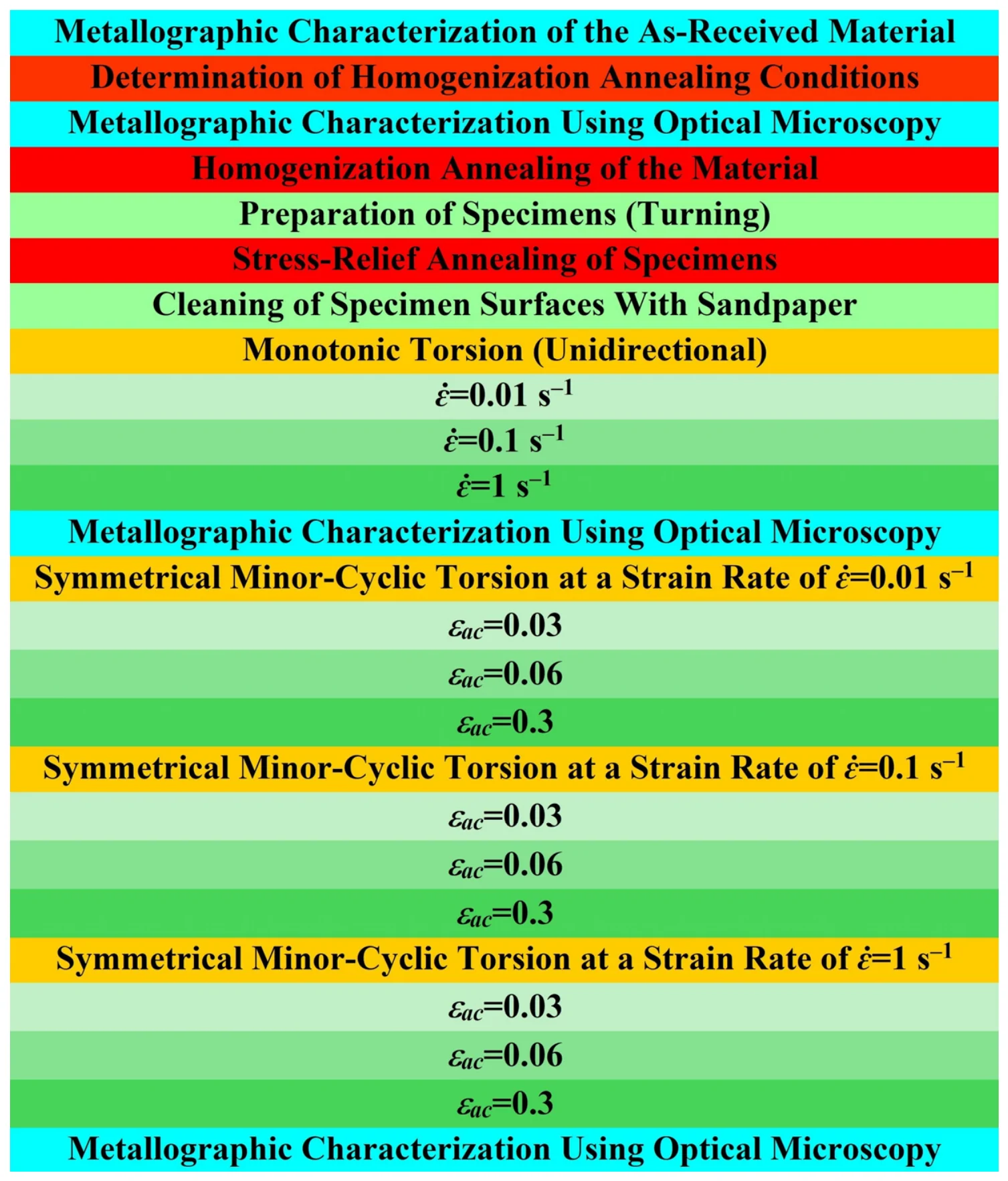
The complete experimental program.

The complete experimental program carried out in this study is presented below in Figure 5.

Each test under specific deformation conditions was repeated three times. When one of the results deviated significantly from the others (by a difference of more than 5%), the test was repeated for verification.

## 3. Results

In this study, stress–strain curves were determined. First, monotonic torsion tests were conducted. Subsequently, cyclic torsion tests with variable strain paths were performed, and their results were compared with those obtained from the monotonic torsion.

### 3.1. Monotonic Torsion Results

**Figure 6:**
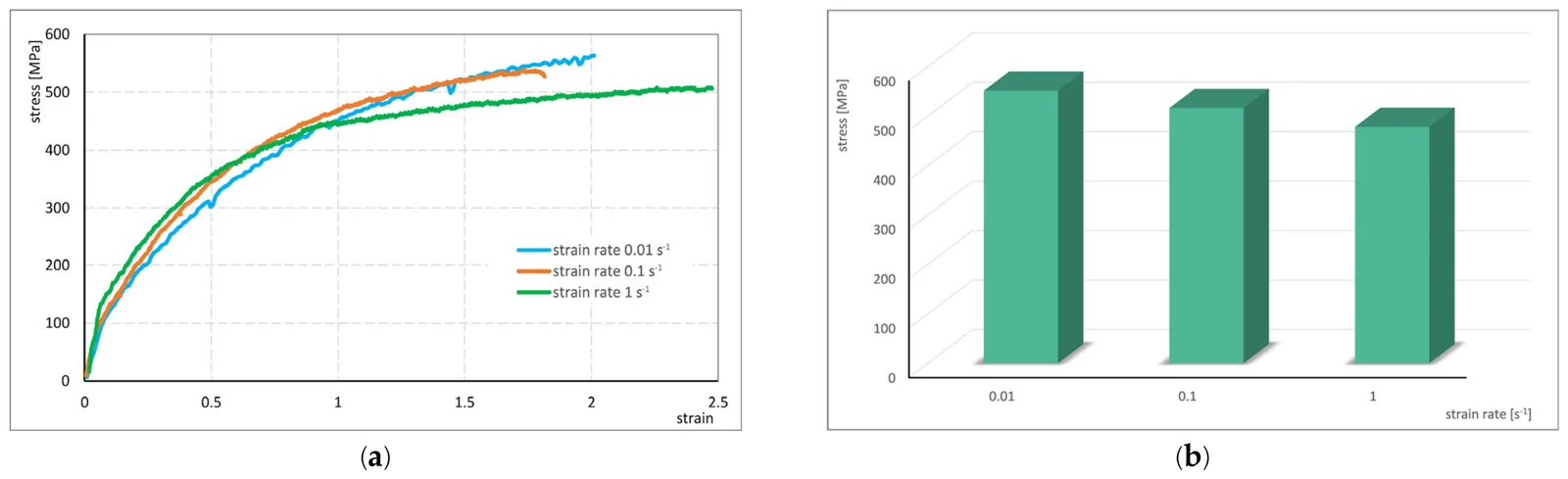
Comparison of (**a**) monotonic torsional stress–strain curves at all investigated strain rates and (**b**) flow stress variation as a function of strain rate for monotonic torsion at room temperature at a strain of 1.8.

The first stage of the investigation was to determine the stress–strain curves in monotonic torsion tests conducted at three selected strain rates: 0.01 s^−1^, 0.1 s^−1^ and 1 s^−1^. The stress–strain curves obtained at room temperature in the monotonic torsion test at various strain rates are presented in Figure 6a. The changes in the maximum flow stress as a function of the strain rate are shown in the bar graph (Figure 6b).

The next stage of the study was to determine the stress–strain curves in cyclic oscillatory symmetrical torsion tests. Strain rates of 0.01 s^−1^, 0.1 s^−1^ and 1 s^−1^ were applied, along with symmetrical strain amplitudes of 0.03, 0.06 and 0.3. These amplitudes resulted—as indicated above—from Equation (2) and from specimen twist amplitudes of angles *ω =* 18° (0.05 revolutions), *ω =* 36° (0.1 revolutions), and *ω =* 180° (0.5 revolutions). Tests with larger strain amplitudes (exceeding 0.5 revolutions) led to very rapid specimen failure after just a few cycles; therefore, the authors decided against them and adopted the amplitude range specified herein.

### 3.2. Cyclic Torsion Results at a Strain Rate of 0.01 s^−1^

**Figure 7:**
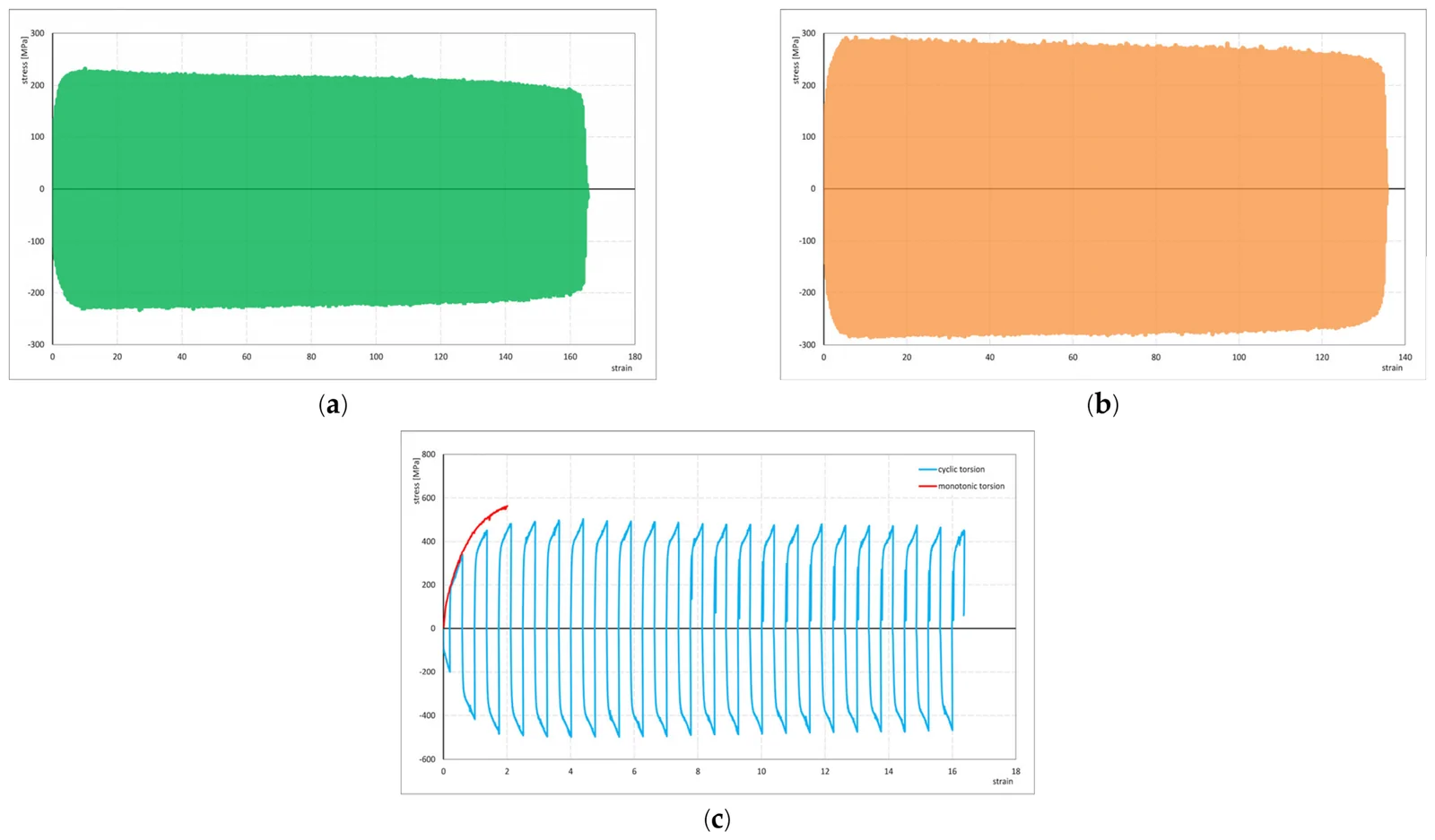
Cyclic torsional stress–strain curves at a strain rate of 0.01 s^−1^: (**a**) strain amplitude of 0.03, (**b**) strain amplitude of 0.06, (**c**) strain amplitude of 0.3 (compared with the monotonic torsion curve).

The results of cyclic torsion at a strain rate of 0.01 s^−1^ and for all three amplitudes are shown in Figure 7, and the detailed numerical results are presented in Table 2.

**Figure 8:**
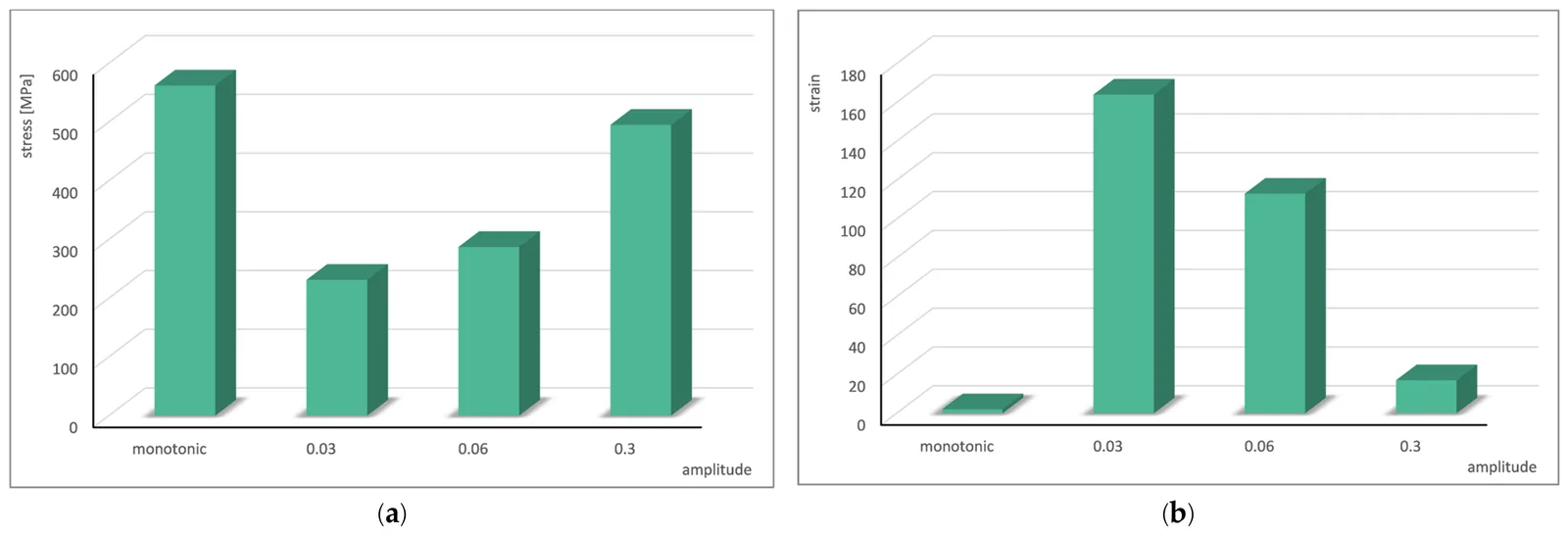
Changes in stress (**a**) and strain (**b**) during cyclic torsion at a strain rate 0.01 s^−1^.

**Figure 9:**
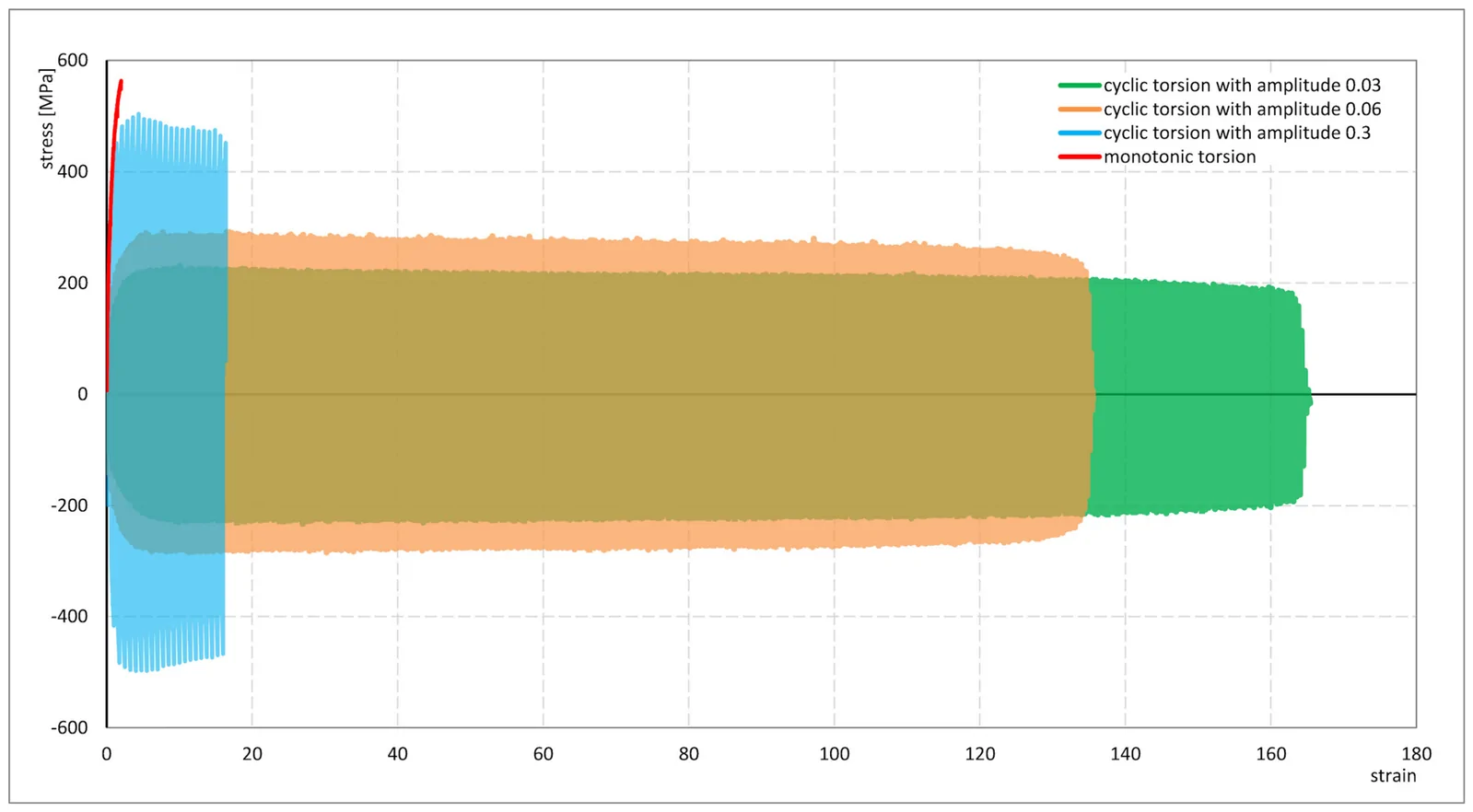
Comparison of cyclic torsion curves at all investigated amplitudes with the monotonic torsion curve, all at a strain rate of 0.01 s^−1^.

To better visualize the obtained results, plots illustrating the changes in stress and strain as a function of amplitude are presented in Figure 8, while Figure 9 shows a comparison of the cyclic torsional stress–strain curves at a strain rate of 0.01 s^−1^ and strain amplitudes of 0.03, 0.06, and 0.3 with the monotonic torsion curve at the same strain rate.

### 3.3. Cyclic Torsion Results at a Strain Rate of 0.1 s^−1^

**Figure 10:**
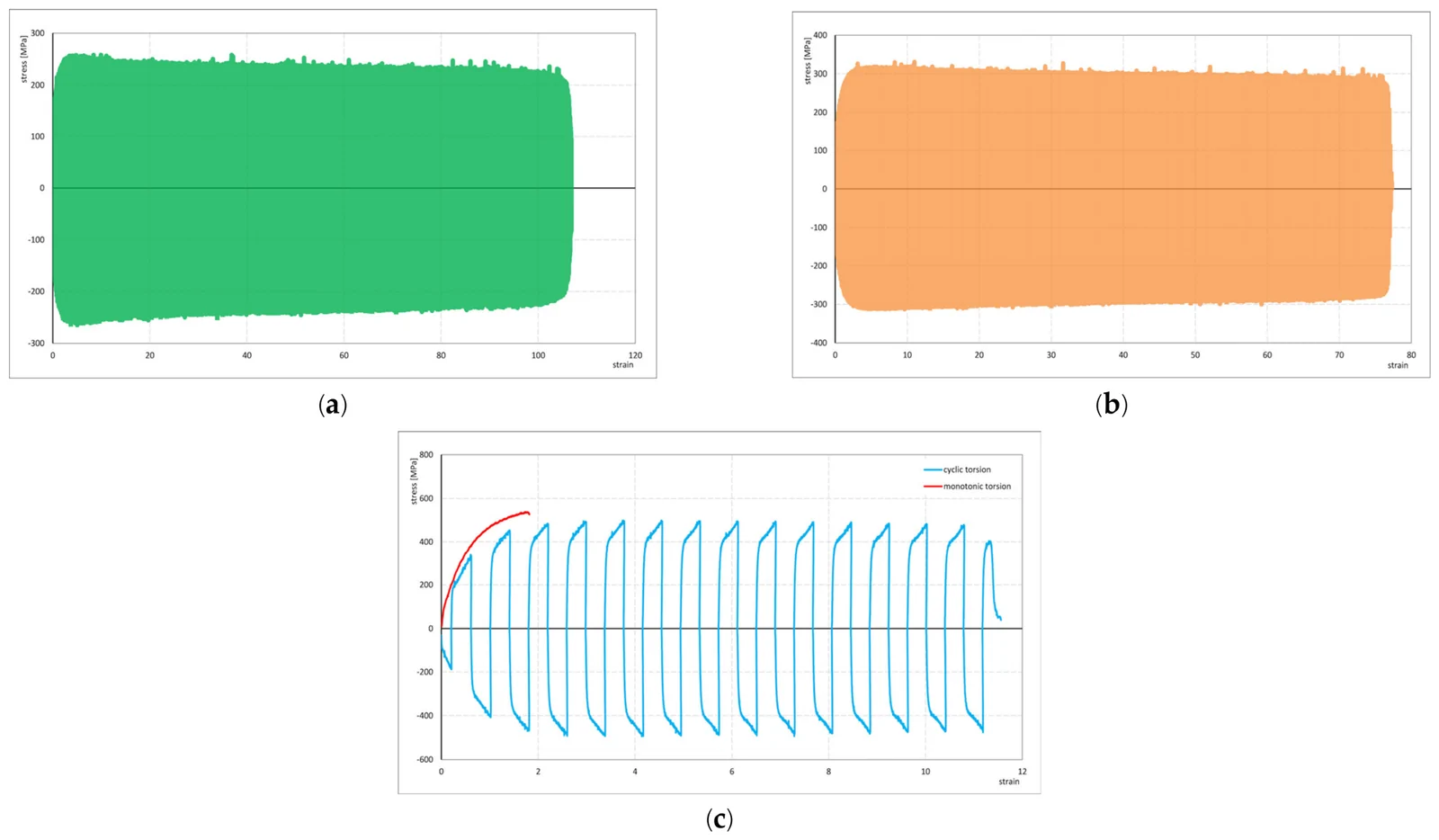
Cyclic torsional stress–strain curves at a strain rate of 0.1 s^−1^: (**a**) strain amplitude of 0.03, (**b**) strain amplitude of 0.06, (**c**) strain amplitude of 0.3 (compared with the monotonic torsion curve).

The next stage of the minor-cyclic investigation was symmetrical torsion at a strain rate of 0.1 s^−1^. The results at a strain rate of 0.1 s^−1^ and for all three amplitudes are shown in Figure 10, and the detailed numerical results are presented in Table 3.

**Figure 11:**
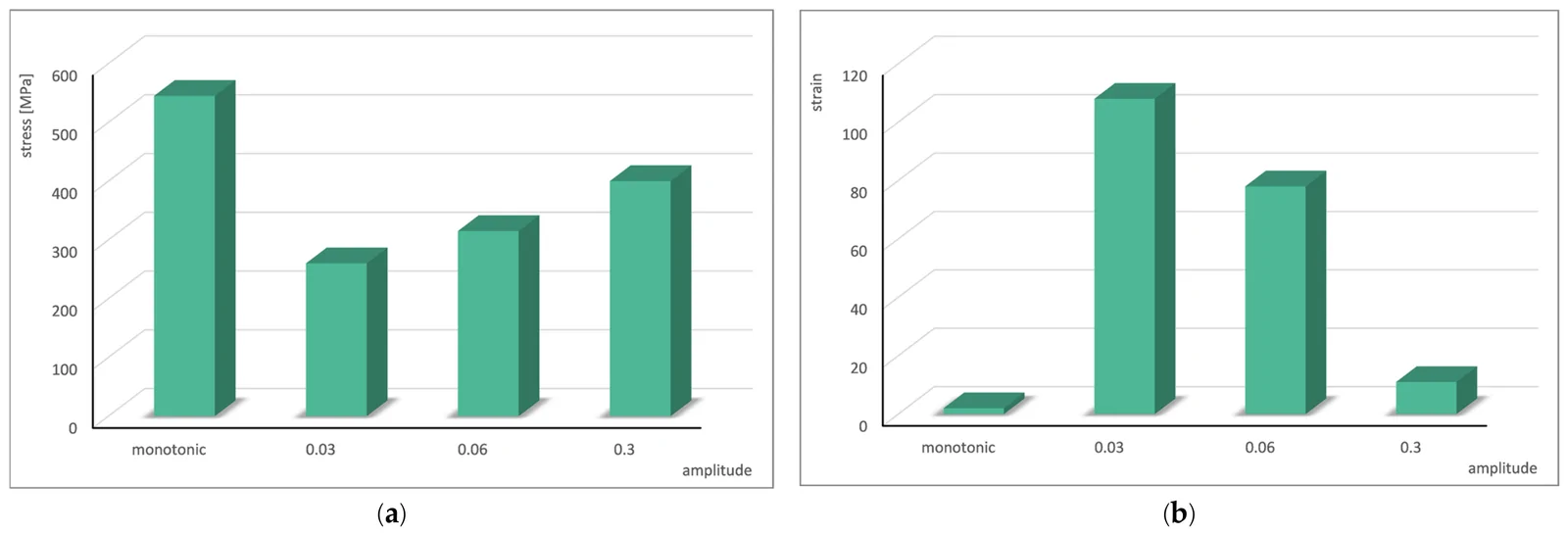
Changes in stress (**a**) and strain (**b**) during cyclic torsion at a strain rate 0.1 s^−1^.

**Figure 12:**
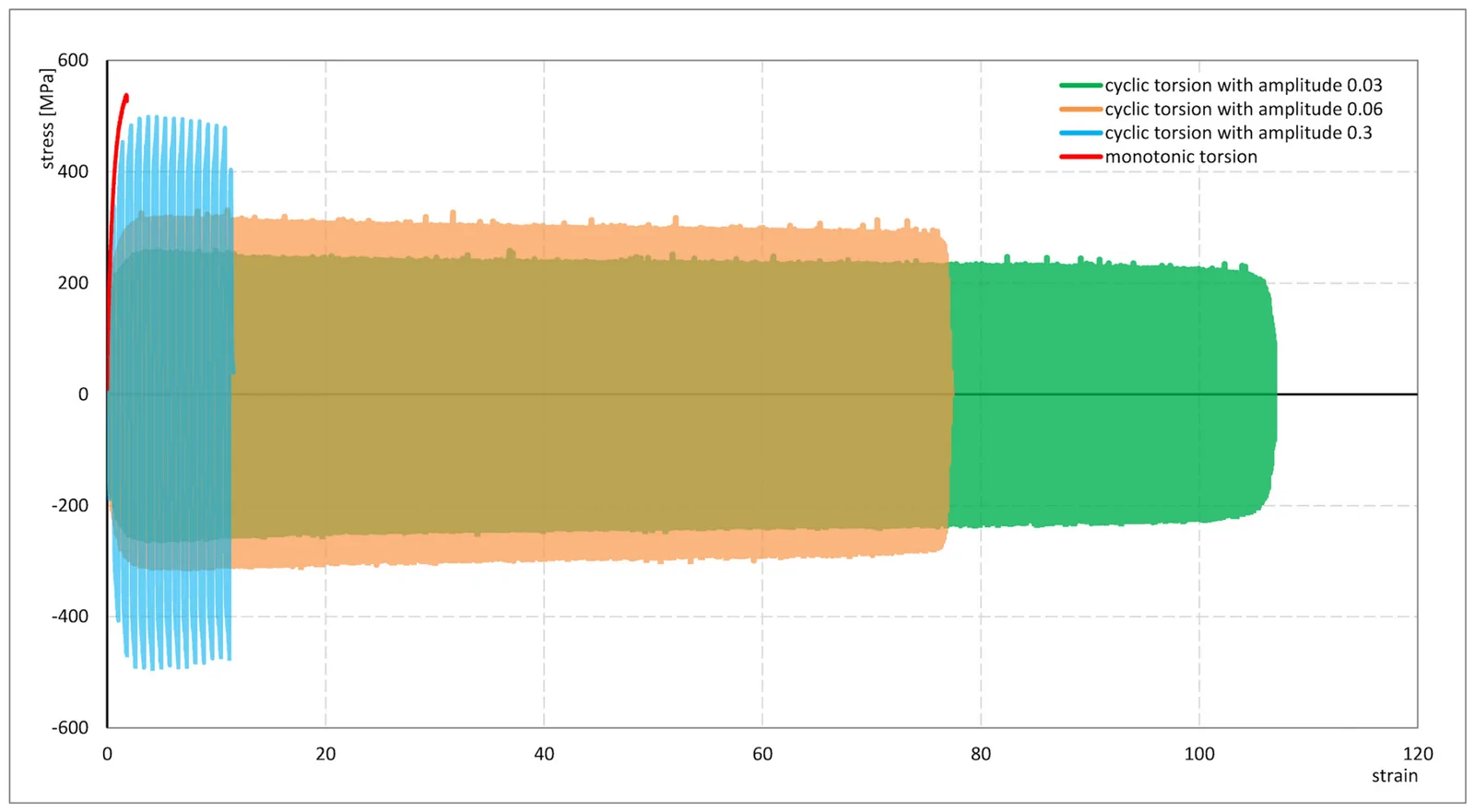
Comparison of cyclic torsion curves at all investigated amplitudes with the monotonic torsion curve, all at a strain rate of 0.1 s^−1^.

To better visualize the obtained results, plots illustrating the changes in stress and strain as a function of amplitude are presented in Figure 11, while Figure 12 shows a comparison of the cyclic torsional stress–strain curves at a strain rate of 0.1 s^−1^ and strain amplitudes of 0.03, 0.06, and 0.3 with the monotonic torsion curve at the same strain rate.

### 3.4. Cyclic Torsion Results at a Strain Rate of 1 s^−1^

**Figure 13:**
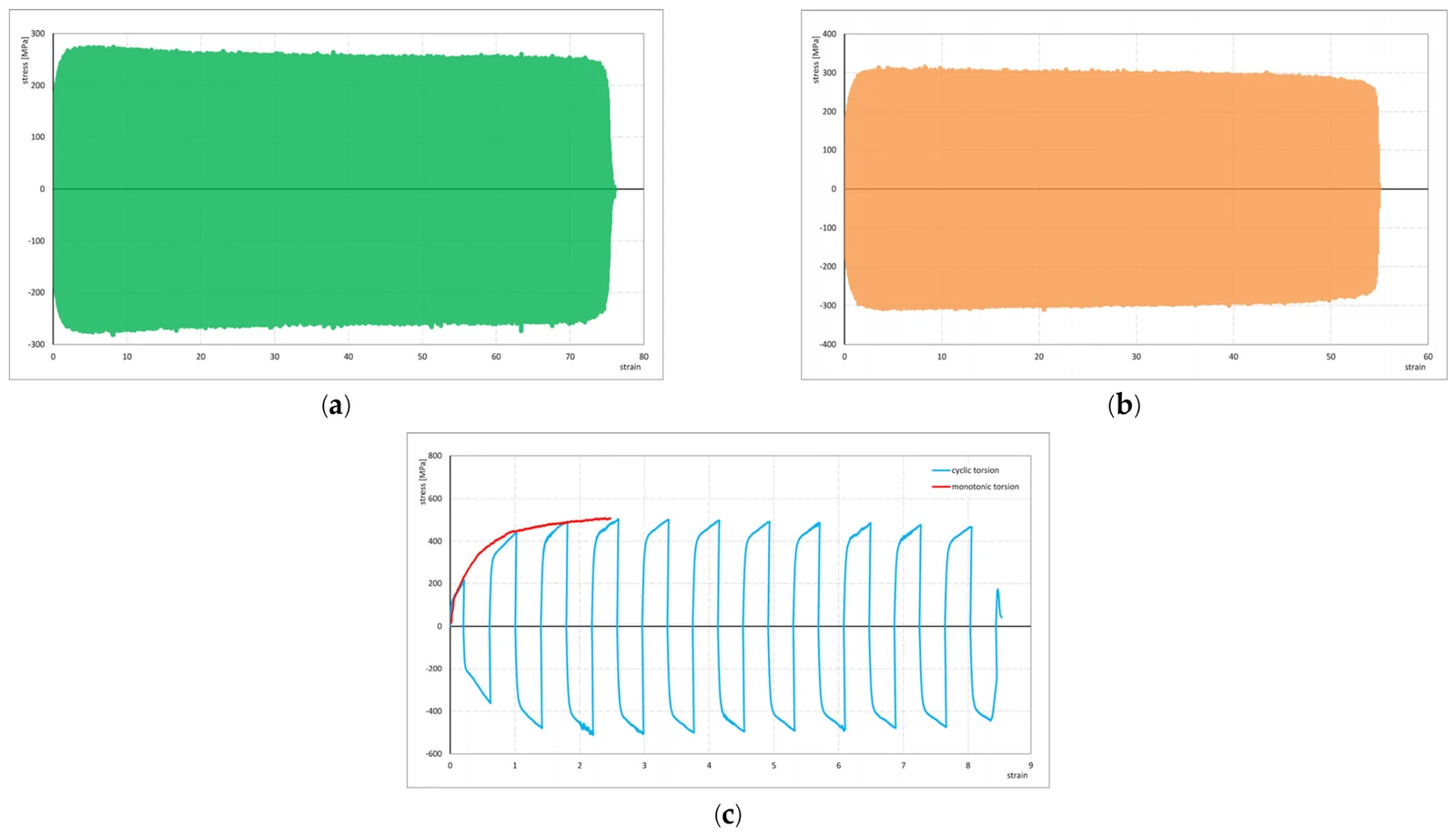
Cyclic torsional stress–strain curves at a strain rate of 1 s^−1^: (**a**) strain amplitude of 0.03, (**b**) strain amplitude of 0.06, (**c**) strain amplitude of 0.3 (compared with the monotonic torsion curve).

The final stage of the investigation was cyclic torsion at a strain rate of 1 s^−1^. Its results are shown in Figure 13, and the detailed numerical results are presented in Table 4.

**Figure 14:**
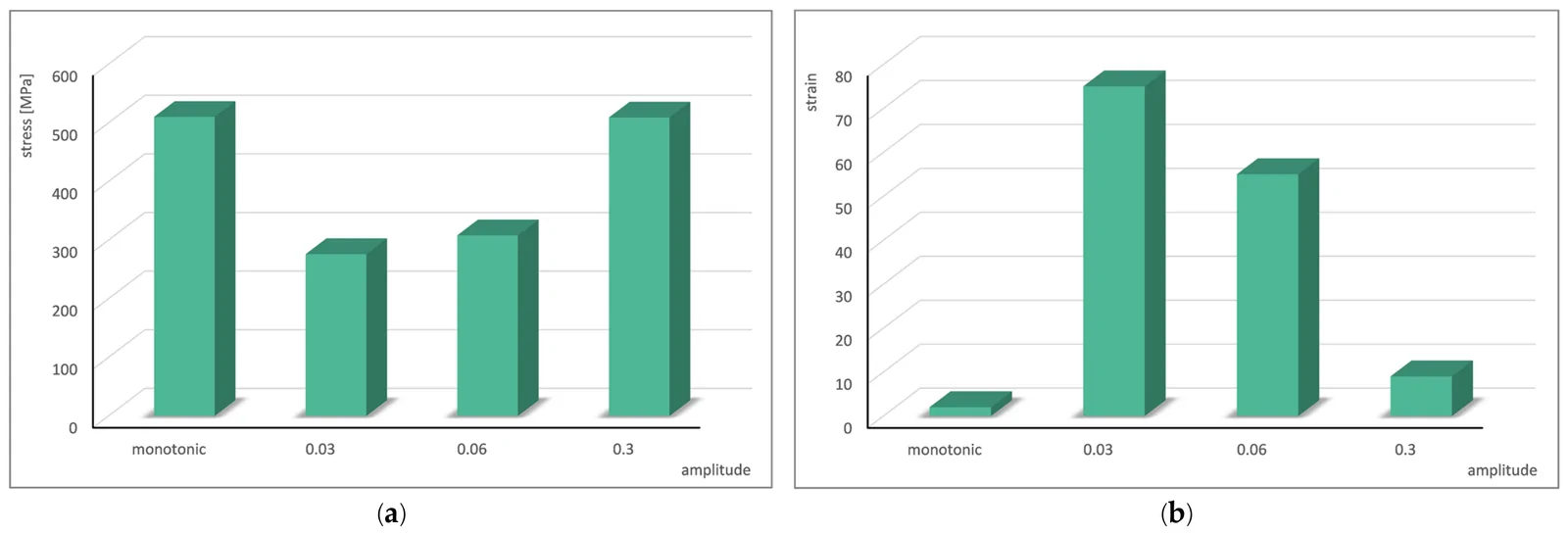
Changes in stress (**a**) and strain (**b**) during cyclic torsion at a strain rate 1 s^−1^.

**Figure 15:**
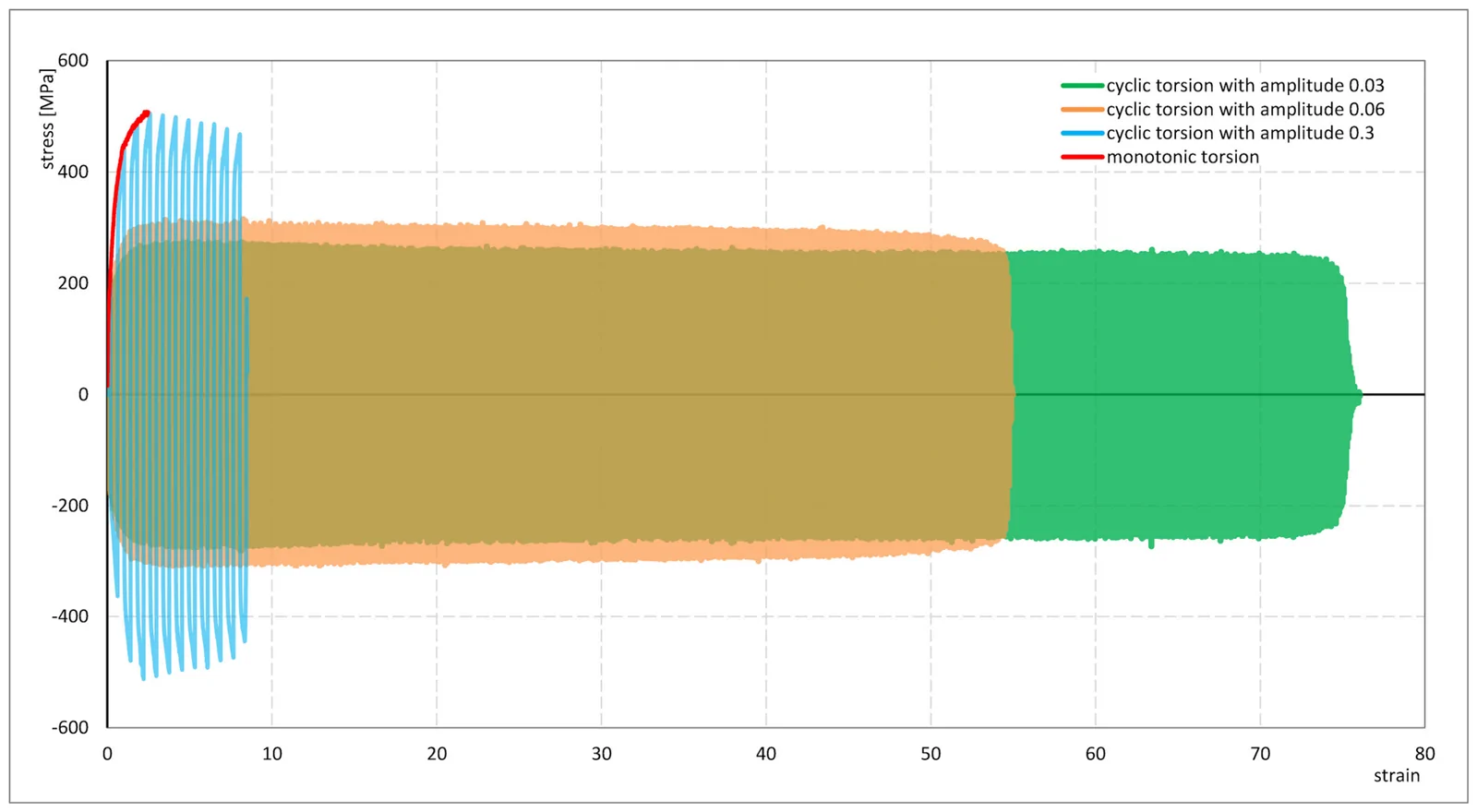
Comparison of cyclic torsion curves at all investigated amplitudes with the monotonic torsion curve, all at a strain rate of 1 s^−1^.

To better visualize the obtained results, plots illustrating the changes in stress and strain as a function of amplitude are presented in Figure 14, while Figure 15 shows a comparison of the cyclic torsional stress–strain curves at a strain rate of 1 s^−1^ and strain amplitudes of 0.03, 0.06, and 0.3 with the monotonic torsion curve at the same strain rate.

## 4. Microstructural Studies

To section the deformed and fractured specimens, a Struers Secotom-50 precision metallographic cutting machine (Struers, Ballerup, Denmark) was used. The sectioned specimens were hot-mounted using a Struers CitoPress-1 mounting press(Struers ApS, Ballerup, Denmark), followed by grinding and polishing on a Struers LaboPol-60 metallographic grinding and polishing machine (Struers ApS, Ballerup, Denmark). Metallographic cross-sections were prepared in a plane parallel to the axis of the specimens. The specimens were etched with a 10% aqueous solution of ammonium persulfate (APS)—(NH_4_)_2_S_2_O_8_—for approximately 2 min. Microstructural observations were carried out using an Olympus GX-51 optical metallographic microscope (Olympus, Tokyo, Japan).

**Figure 16:**
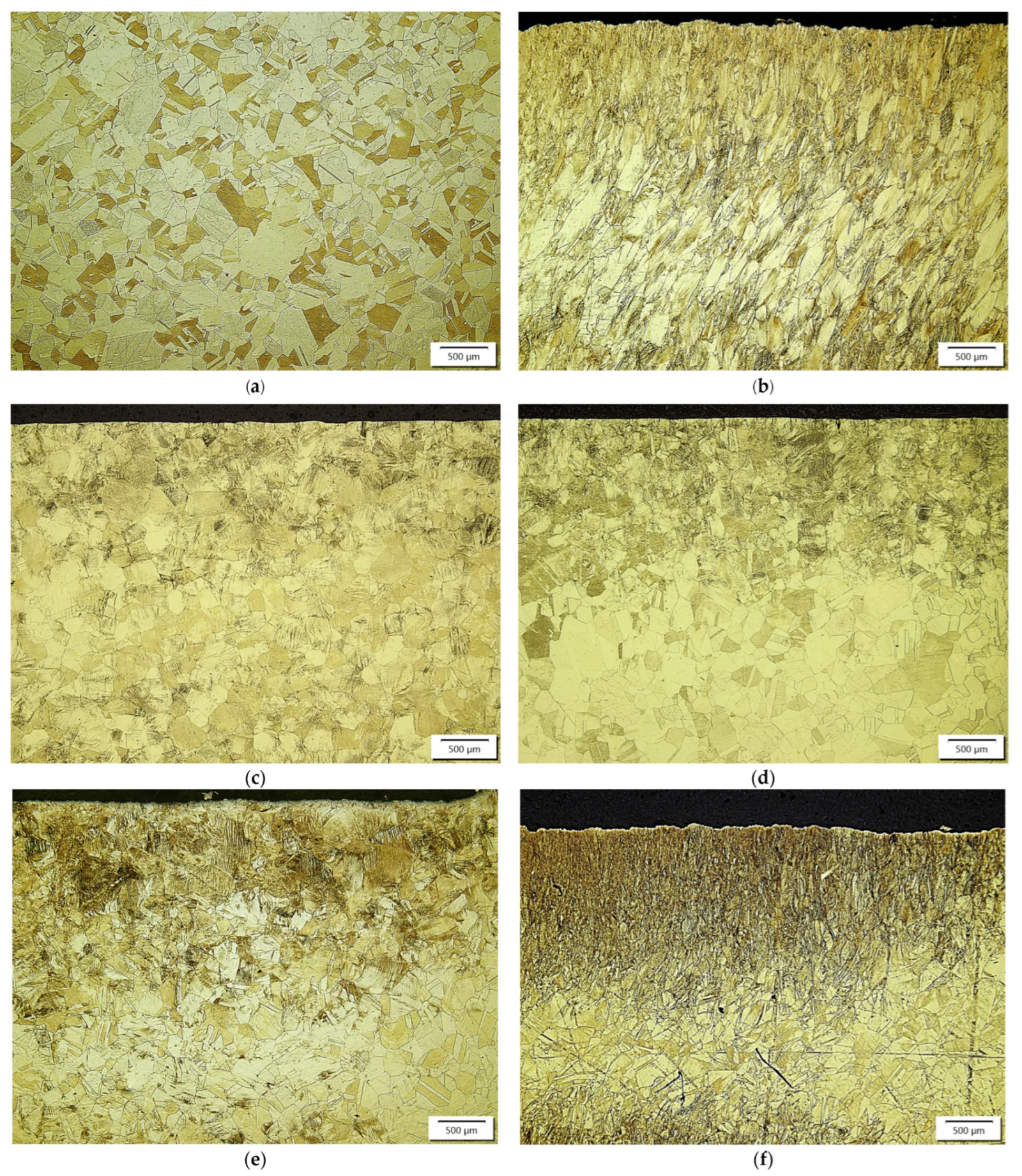
Microstructures of the investigated CuZn30 brass: (**a**) undeformed material, (**b**) after monotonic torsion at a strain rate of 1 s^−1^, (**c**) after cyclic torsion at a strain rate of 0.01 s^−1^ and an amplitude of 0.03, (**d**) after cyclic torsion at a strain rate of 0.1 s^−1^ and an amplitude of 0.03, (**e**) after cyclic torsion at a strain rate of 1 s^−1^ and an amplitude of 0.03, (**f**) after cyclic torsion at a strain rate of 1 s^−1^ and an amplitude of 0.3.

The analysis was performed in accordance with ASTM E112 [21], and the appropriate grain size numbering G was given. Figure 16 presents selected micrographs of the investigated material, followed by their detailed analysis below.

The microstructure of the undeformed CuZn30 brass prepared for examination is shown in Figure 16a. It is a microstructure typical of annealed α-brasses. It is characterized by a morphology of equiaxed grains of the α-phase solid solution, featuring rectilinear boundaries, with a very high density of annealing twins. The colour contrast (shades of yellow and brown) results from the anisotropic etching of grains with different crystallographic orientations. No presence of slip bands or deformed grains was observed. The grain size is varied: the structure exhibits moderate bimodality (distinctly different grain size ranges) and anisometry. Besides the dominant medium-sized grain fractions (70–120 µm), distinct, single large grains with diameters exceeding 200–250 µm and clusters of finer grains (<40 µm) are visible. The estimated average equivalent grain diameter is approximately 90–130 µm, which according to the ASTM E112 standard corresponds to grain size number G2.5–G4.

Figure 16b shows the microstructure after monotonic (unidirectional) torsion at a strain rate of 0.1 s^−1^. Distinct material flow in the deformation direction is visible, along with a pronounced morphological texture (note: not to be confused with crystallographic texture, which is invisible under an optical microscope) and numerous slip bands. Compared to the undeformed microstructure (Figure 16a), the unidirectional torsion test introduced a strong deformation gradient into the investigated material, increasing from the specimen core toward its surface (top of the image), which fundamentally alters the geometric parameters of the grains. A clear gradient distribution is apparent: the closer to the outer surface of the specimen, the higher the strain degree, the more flattened the grains become, and the densities of shear bands and deformation twins grow rapidly. All grains underwent rotation (twisting) and a distinct inclination at an angle of approx. 45–60° relative to the specimen axis, consistent with the local direction of maximum shear stresses during torsion. The grain size distribution is highly heterogeneous, exhibiting a large scatter in grain length. Inside most of them, a dense network of slip bands developed, which is a direct consequence of the low SFE of the CuZn30 brass. Due to the strong grain anisometry (elongation), their size is not determined here by a single equivalent diameter, but by two dimensions. The grain width (transverse to the flow line) is approx. 20–50 µm, while their length (along the shear direction)—due to grain inclination and elongation—ranges from 150 to over 350 µm. Dense shear and deformation bands are visible inside the primary grains, partitioning them into subgrains with sizes well below 20–50 µm.

The remaining four micrographs, shown in Figure 16c–f, present the microstructures of the investigated material subjected to minor-cyclic torsion under different conditions. In the case of bilateral—and thus cyclic—torsion involving a change in the deformation direction, the geometric characteristics of the grains undergo a secondary alteration due to the phenomenon of strain path reversal. In contrast to monotonic torsion (Figure 16b), where a strong morphological (geometric) texture occurs and the grains are drastically elongated along the shear lines, the grains in this case predominantly maintain a nearly equiaxed shape. Indeed, the cyclic deformation causes the geometric changes in grain shape to counteract one another, preventing the formation of a macroscopic morphological texture. The alternating change in the torsion direction causes the grains—initially flattened and inclined in one direction—to return to a geometry closer to more regular, roughly equiaxed polygons.

A significant plastic deformation of the material in the subsurface region is visible in all these figures. Conversely, near the specimen axis, the material is only slightly deformed, exhibiting a microstructure roughly similar to the initial state (the undeformed material), where only minor grain deformation occurs. This type of deformation results from the non-uniform strain distribution across the specimen cross-section during torsion testing, where the strain reaches its maximum in the outer layers and its minimum near the specimen axis. Numerous slip bands are visible in the outer part of the specimen, leading to substantial microstructure deformation. Consequently, a distinct deformation gradient is clearly revealed after bilateral torsion—the closer to the surface (the top of the micrograph), the higher the degree of material damage, which is manifested by a more pronounced matte appearance of the grains, a higher defect density, and a denser network of intra-granular microstructures.

Figure 16c shows the microstructure after cyclic torsion at a strain rate of 0.01 s^−1^ and a strain amplitude of 0.03, which represent the lowest strain rate and the smallest amplitude used in this study. Inside most of the grains (particularly in the subsurface zone), a high density of linear and band-like features is visible. The low SFE of the CuZn30 brass promotes the intensive generation of slip bands, which intersect at different angles depending on the crystallographic orientation of the individual grains. Twins are also visible, along with wide, straight bands that constitute primary annealing twins.

The microstructure has retained the geometric equiaxed characteristics of the matrix, allowing for the application of the standard procedure for estimating the average grain size. The dominant fraction consists of medium and large grains, with diameters most frequently falling within the 70–130 µm range. A few isolated larger grains and smaller regions pinned at twin boundaries are also apparent. The estimated average equivalent grain diameter falls within the range of approx. 85–110 µm—according to the ASTM E112 standard, this geometric size corresponds to grain size number G3–G3.5.

Figure 16d shows the microstructure after cyclic torsion at a strain rate of 0.1 s^−1^ and a strain amplitude of 0.03. Compared to the microstructure described above, the tenfold increase in the strain rate drastically restricts the time available for local dynamic recovery processes, causing the deformation energy to accumulate within the material in an abrupt and intense manner. In the bottom half of the image (near the specimen axis), the grains have retained their equiaxed shape, featuring distinct boundaries and wide annealing twins. The fact that the matrix grains are not elongated under a significantly higher strain rate confirms that during cyclic torsion, the geometric changes in grain shape counteract each other in every cycle, thereby preventing the formation of a morphological texture.

In the subsurface zone (a band spanning approximately 300 µm from the specimen edge), the defect density is exceptionally high. The low SFE of the CuZn30 brass subjected to rapid cyclic shearing induced a rapid multiplication of slip bands and deformation micro-twins. This region exhibited more intense etchant absorption, resulting in a darker, matte, and heavily defected appearance where the primary grain boundaries undergo local blurring.

The higher strain rate at room temperature does not lead to dynamic recrystallization; therefore, the matrix grain size remains geometrically close to the initial state, although the grain interiors are more severely damaged. The structure is dominated by a medium-sized grain fraction, with diameters mostly falling within the 70–120 µm range. Moderate bimodality is apparent—alongside the dominant medium fraction, a single large grain is visible in the bottom-left part of the micrograph, and clusters of finer grains (<40 µm) are present at the grain boundaries. The estimated average equivalent grain diameter for this entire microstructure is approximately 85–110 µm. According to the ASTM E112 standard, this geometric size corresponds to grain size number G3–G3.5.

Figure 16e shows the microstructure after cyclic torsion at a strain rate of 1 s^−1^ and a strain amplitude of 0.03, representing the highest strain rate and the smallest amplitude used in this study. The micrograph reveals a further degree of material degradation and defect accumulation, reflecting the change in the test parameters.

A distinct deformation gradient from the subsurface region to the specimen core is still apparent. The density of deformation substructures is highest in the subsurface layer. The dark, heavily defected band extending from the upper edge of the specimen is visibly wider and more saturated with defects than in the previous Figure 16d. It already reaches a depth of over 500–600 µm from the specimen edge.

Dense shear bands propagating in two mutually intersecting directions are visible, between which a strain path alteration occurred. Strong activation of complex slip and twinning systems occurs inside the grains, causing internal fragmentation of the microstructure. Due to the high density of shear bands and deformation twins, this region has undergone very intense chemical etching.

In the zone just below the specimen edge, it is apparent that the densely spaced shear bands physically intersect the primary grains, partitioning them into fine subgrains. This phenomenon initiates the process of mechanical structural refinement induced by cyclic shearing.

Even in the middle and bottom parts of the image (near the specimen axis), the grain interiors are no longer as clean as before. An exceptionally dense network of parallel and intersecting slip bands is visible inside almost every grain. In many grains, slip has been activated in multiple crystallographic systems simultaneously, which is a direct consequence of the low SFE of the investigated brass at a higher degree of accumulated plastic strain.

Despite the drastic increase in the intra-granular defect density, the matrix in the bottom part of the image still retains the characteristics of an equiaxed microstructure—there is no unambiguous, macroscopic elongation of the grains in a single direction, which confirms the bilateral nature of the plastic deformation. The geometric outline of the grains in the depth of the material allows determining that the dominant fraction falls within the range of 70–120 µm. The microstructure still exhibits features of moderate bimodality, with isolated large grains in the matrix core and highly fragmented areas in the subsurface zone. The estimated average equivalent grain diameter of the matrix falls within the range of approx. 85–110 µm, which corresponds to grain size number G3–G3.5 according to the ASTM E112 standard. However, it must be clearly noted that in the subsurface zone (top of the image), the effective size of the subgrains has decreased significantly below the initial value due to the dense network of bands. A dense, intersecting network of microbands and shear bands has developed inside the grains, partitioning individual grains into local domains with sizes even below 20 µm.

Figure 16f shows the microstructure after cyclic torsion at a strain rate of 1 s^−1^ and a strain amplitude of 0.3, representing the highest strain rate and the largest amplitude used in this study. The most advanced stage of microstructural degradation among all previously analysed states is evident. The combination of a high strain rate and a very large strain amplitude of 0.3 (which translates to a rotational amplitude of as much as 180°) induced deep metallographic changes. A tenfold increase in the cyclic torsion amplitude led to a drastic strain accumulation in the subsurface layers, triggering an effect of profound refinement and fragmentation of the microstructure. The micrograph reveals a very large microstructural gradient: the image is clearly divided into two zones, with an extremely expanded, dominant subsurface zone. The dark, heavily defected subsurface region expanded drastically and occupies nearly half the height of the entire microstructure presented here. It is an intensely etched band characterized by an exceptionally high density of dislocations, shear bands, and deformation micro-twins. The substantial cycle amplitude forced the overlapping of intersecting shear bands in multiple directions within the material. This led to deep fragmentation—partitioning the primary grains into numerous subgrain blocks with a high angle of misorientation, a phenomenon analogous to structural refinement in severe plastic deformation (SPD) processes.

At such a high strain amplitude, blurring of the primary grain boundaries occurs: in the outer (subsurface) layer, the former grain boundaries cease to be distinct. The classical, straight grain boundaries of the single-phase α solid solution and the straight boundaries of the annealing twins underwent severe waving or complete annihilation, making them very difficult to unambiguously identify under an optical microscope. The microstructure assumed the form of highly sharpened, vertically and obliquely oriented, densely packed band structures (shear bands and packages of micro-twins) with a very high density of defects. These band structures formed a local morphological texture of a fibrous nature. This phenomenon did not occur at a smaller amplitude.

Defect accumulation also affected the matrix core: even close to the specimen axis, the grain interiors are highly distorted, and it is difficult to find a grain free of defects. The low SFE of the CuZn30 brass led to the formation of an exceptionally dense, almost continuous intra-granular network—slip bands intersect at multiple angles, completely filling the grain volume.

Due to such a significant degree of plastic deformation, determining the traditional matrix grain size for the entire micrograph is impossible because the structure is highly non-uniform and locally fragmented. Closer to the specimen axis, where the outlines of the primary grains are still visible, the dominant geometric fraction falls within the range of approximately 70–120 µm. The estimated average equivalent grain diameter of the primary matrix grains is approximately 85–110 µm, which formally corresponds to grain size number G3–G3.5 according to the ASTM E112 standard. However, it must be strictly emphasized that the stated G parameter cannot be taken literally here, as it refers exclusively to the outline of the primary matrix grains in the core. In reality, under the influence of cyclic torsion with the parameters applied here, dense shear bands partitioned the material in the subsurface zone into subgrains with an effective size well below 5–15 µm (G8.5–G11.5—a fine-grained structure, locally tending toward an ultrafine-grained state). As the distance from the surface toward the specimen axis increases, primary grains with dimensions of approximately 40–80 µm (G4–G6) become visible, though they are densely saturated with internal defects.

To summarize the findings from the microstructural characterization, based on the analysis of the presented CuZn30 brass microstructures (ranging from the annealed state through monotonic torsion to bilateral torsion conducted at various strain amplitudes and strain rates), it is possible to determine the influence of the strain path on grain morphology, as well as the effects of the strain rate and amplitude on the degree of structural refinement.

Monotonic torsion drives an anisotropic change in grain shape, where the grains undergo severe elongation along with a distinct rotation and inclination at a specific angle relative to the specimen axis. Conversely, cyclic torsion restricts the development of geometric grain anisometry. The reversal of the shearing direction allows the grains to maintain a more equiaxed shape compared to monotonic torsion, despite the accumulation of high plastic strain.

Variations in the strain rate, while maintaining the same strain amplitude, do not fundamentally affect the grain size in the matrix core. In contrast, an increase in the amplitude rapidly intensifies the fragmentation process, leading to the blurring of the primary grain boundaries and the formation of a fine-grained or even an ultrafine-grained structure. Furthermore, increasing the amplitude drastically expands the zone of intense deformation deeper into the material, establishing a pronounced, deep microstructural gradient from the outer surface to the specimen axis.

## 5. Discussion

At strain rates higher than those at which creep occurs, strain hardening processes dominate, and the CuZn30 brass exhibits no distinct strain rate sensitivity at room temperature. This was demonstrated by the results of monotonic (unidirectional) torsion tests presented in Figure 6: a tenfold increase in the strain rate induces only a very minor change in the stress value. Therefore, in the investigations conducted at room temperature, the strain rate sensitivity exponent *m* was neglected due to its exceedingly low value under these temperature conditions. The strain hardening exponent *n* was equal to 0.435.

During monotonic torsion, the strains vary slightly with the change in strain rate—within the range from approximately 1.8 to 2.5. Up to a strain value of about 0.6, the highest stress was achieved for the highest strain rate of 1 s^−1^, whereas the lowest was recorded for the rate of 0.01 s^−1^. At strains higher than 1, the lowest stress value was obtained for the strain rate of 1 s^−1^, which was most likely induced by a temperature increase within the specimen deformed at the highest of the applied rates (adiabatic heating).

In cyclic tests (fully reversed torsion) at a strain rate of 0.01 s^−1^ (Figure 7 and Figure 9 and Table 2) with the two smaller amplitudes, the increase in the limit strain was remarkably large: over 80-fold for the amplitude of 0.03 and nearly 70-fold for the amplitude of 0.06. Such large strains were achieved at a significantly lower flow stress. For the test with an amplitude of 0.03, the maximum stress was approximately 230 MPa, which was nearly 2.5 times lower than the maximum stress in the monotonic test, and it remained at a similar level throughout the entire duration of the test. A similar effect was also obtained during the test with an amplitude of 0.06, for which the flow stress was nearly twice as low as in the monotonic test, amounting to approximately 290 MPa. In the case of the test with a strain amplitude of 0.3, the maximum strain was slightly over 16, which represents roughly eight times the value obtained in the monotonic test. In contrast, the stress was distinctly higher than in the other cyclic tests.

To summarize the above results in percentage terms, compared to monotonic tests, minor-cyclic torsion at a strain rate of 0.01 s^−1^ resulted in:

- For a strain amplitude of 0.03: an 8100% increase in strain and a 59% decrease in stress;
- For a strain amplitude of 0.06: a 6800% increase in strain and a 49.1% decrease in stress;
- For a strain amplitude of 0.3: a 720% increase in strain and a 12.2% decrease in stress.

During cyclic torsion at a strain rate of 0.1 s^−1^ (Figure 10 and Figure 12 and Table 3) and the smallest strain amplitude, the stress stabilized at a level of approximately 260 MPa, whereas for the amplitude of 0.06, it leveled off at about 315 MPa. When the largest amplitude of 0.3 was applied, the stress level was the highest, amounting to around 500 MPa, which is only slightly lower compared to the stress during monotonic torsion. In the tests conducted at a strain rate of 0.1 s^−1^ with the two smaller amplitudes, the increase in strain—just as with the strain rate of 0.01 s^−1^—was remarkably large, being approximately 60-fold and 40-fold, respectively. For the amplitude of 0.3, the strain reached about 11, which represents roughly six times the maximum value obtained in the monotonic test.

To summarize the results in percentage terms, compared to monotonic torsion, minor-cyclic torsion at a strain rate of 0.1 s^−1^ resulted in:

- For a strain amplitude of 0.03: a 5867% increase in strain and a 50% decrease in stress;
- For a strain amplitude of 0.06: a 4209% increase in strain and a 39.4% decrease in stress;
- For a strain amplitude of 0.3: a 508% increase in strain and a 3.85% decrease in stress.

In all tests conducted at a strain rate of 1 s^−1^ across the applied amplitudes (Figure 13 and Figure 15 and Table 4), the smallest increase in deformability relative to the monotonic test was achieved, which was significantly lower than in the case of the tests performed at strain rates of 0.01 s^−1^ and 0.1 s^−1^. For the individual amplitudes, the strain reached values of approximately 75, 55, and 9, respectively. In turn, the flow stress stabilized at a level very close to the stress observed in the minor-cyclic torsion tests at a strain rate of 0.1 s^−1^, amounting to approximately 280 MPa, 310 MPa, and 510 MPa, respectively.

Presenting these as percentage changes, compared to monotonic torsion, minor-cyclic torsion at a strain rate of 1 s^−1^ resulted in:

- For a strain amplitude of 0.03: a 2936% increase in strain and a 45.9% decrease in stress;
- For a strain amplitude of 0.06: a 2127% increase in strain and a 39.6% decrease in stress;
- For a strain amplitude of 0.3: a 244% increase in strain and virtually no change in stress.

It is worth noting that in the tests conducted at all applied strain rates, the stress for the amplitude of 0.3 was always distinctively higher than for the two smaller amplitudes. This is caused by the larger strain in a single cycle compared to the tests with the smaller amplitude. Furthermore, Figure 7, Figure 10 and Figure 13 reveal a clear material softening in the stress–strain curve for the test with an amplitude of 0.3: after reaching the maximum stress value (e.g., approximately 495 MPa at a strain of 4 for the test at a strain rate of 0.01 s^−1^) a noticeable drop occurs, followed by stabilization at a lower level (to a level of about 470 MPa within the strain range of roughly 9–15 for the test at a strain rate of 0.01 s^−1^).

Metals and alloys with a low stacking fault energy—such as CuZn30 brass, among others—are characterized by strong strain hardening and high flow stress values due to the high propensity of dislocations to dissociate, which restricts the dynamic recovery process. Applying a forced strain path change significantly affects the dislocation structure, increasing the dislocation annihilation rate and thereby intensifying the progress of dynamic recovery, which substantially eliminates the effects of strain hardening. This is caused by the material’s response to the forced strain path change applied in the tests, which depends on the angle between the displacement velocity vectors. Thus, the behaviour of the investigated material, as presented in the stress–strain curves for various strain rates and amplitudes, stems from the fact that subsequent cycles with a large amplitude (0.3) intensify the dynamic recovery process, yet lead to earlier failure of the specimen. Tests with a smaller strain amplitude (0.03 and 0.06) do not induce such intense dynamic recovery, but they allow large strains to be achieved. At larger strains, no decrease in the flow stress occurs; instead, it reaches a near-steady state. At the cold working temperature, forcing a 180° change in the deformation direction can lead to a reduction in the yield stress in subsequent strain cycles. Such material behaviour leads to an enhancement of its deformability.

**Figure 17:**
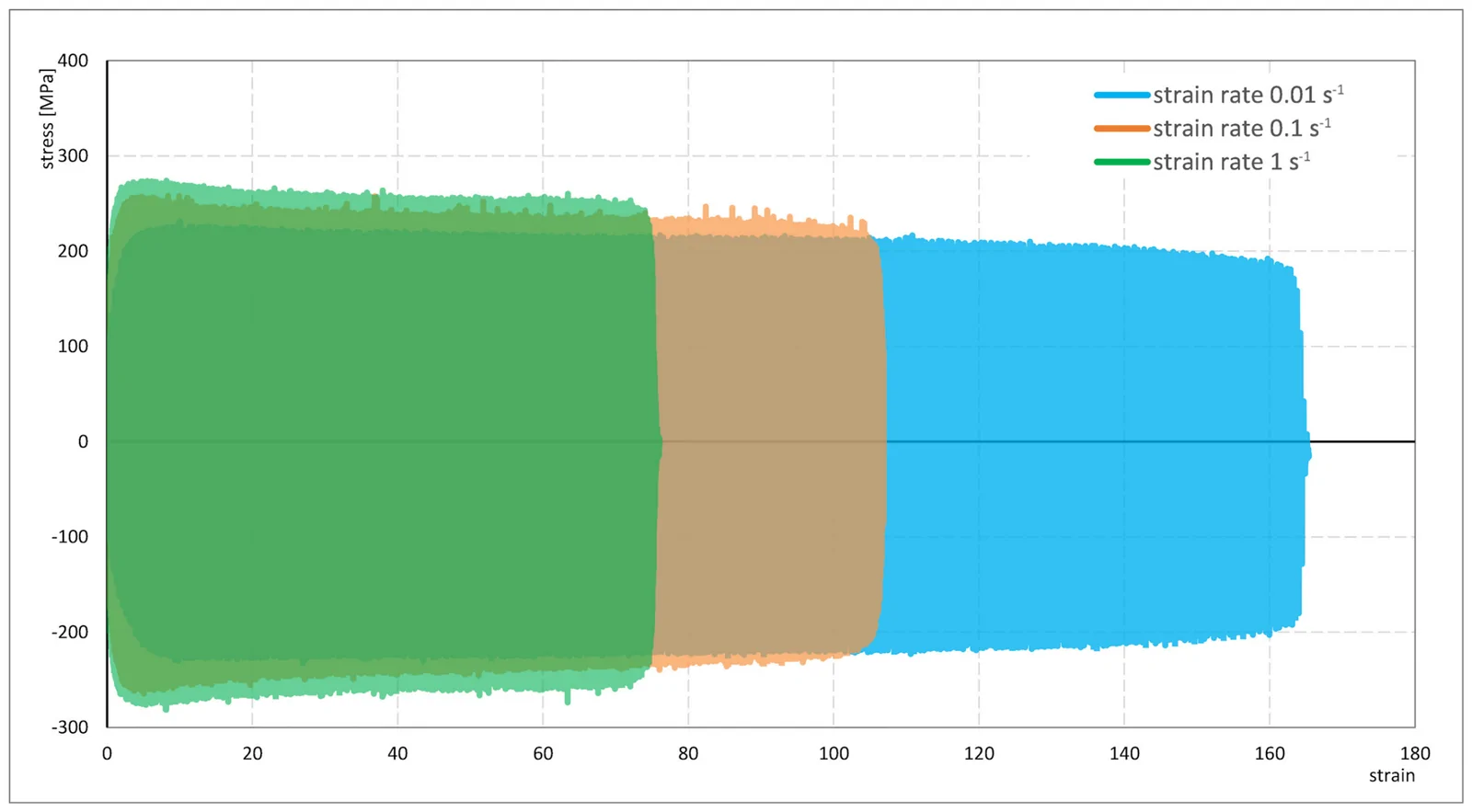
Comparison of cyclic torsion curves at a strain amplitude of 0.03 and all investigated strain rates.

**Figure 18:**
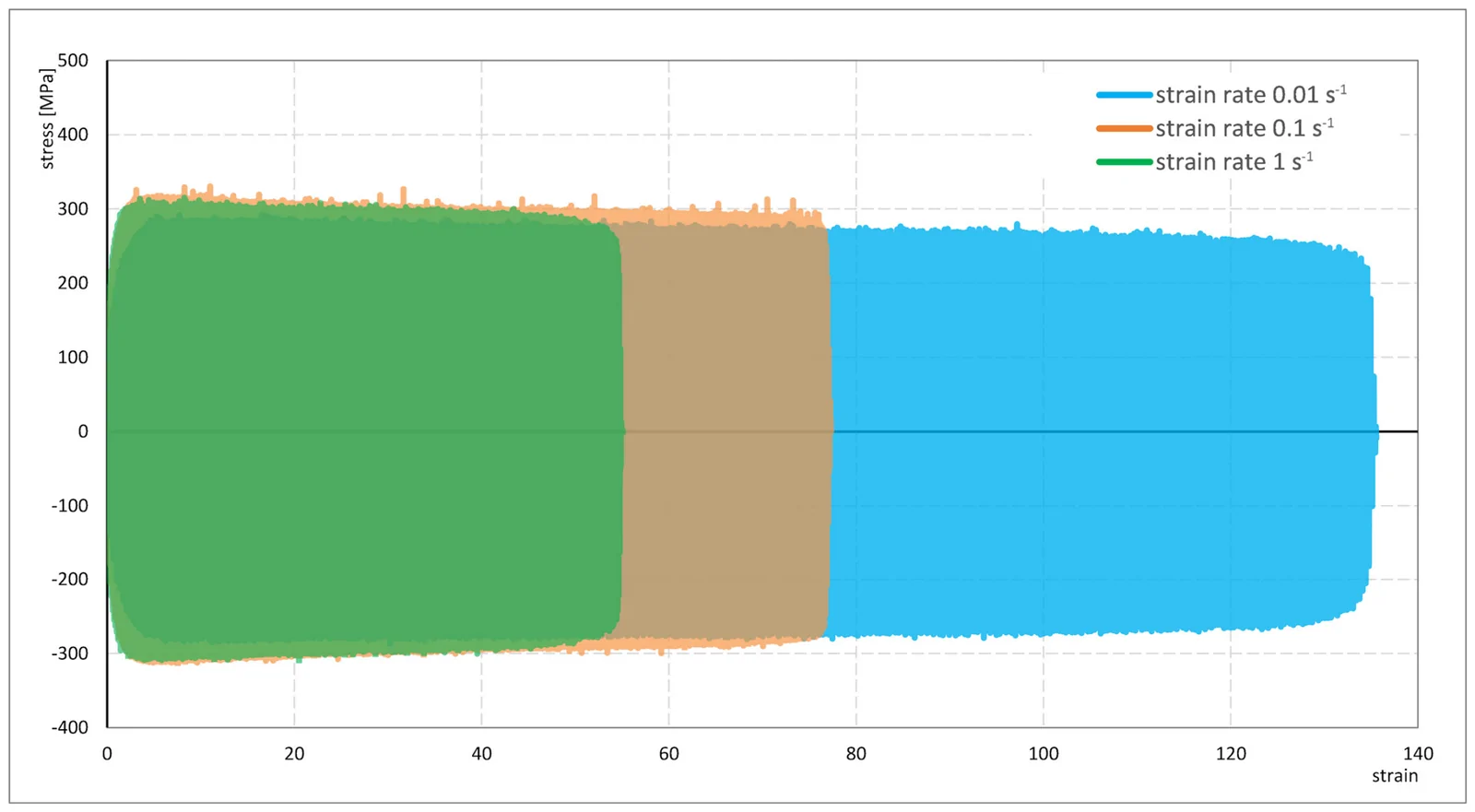
Comparison of cyclic torsion curves at a strain amplitude of 0.06 and all investigated strain rates.

**Figure 19:**
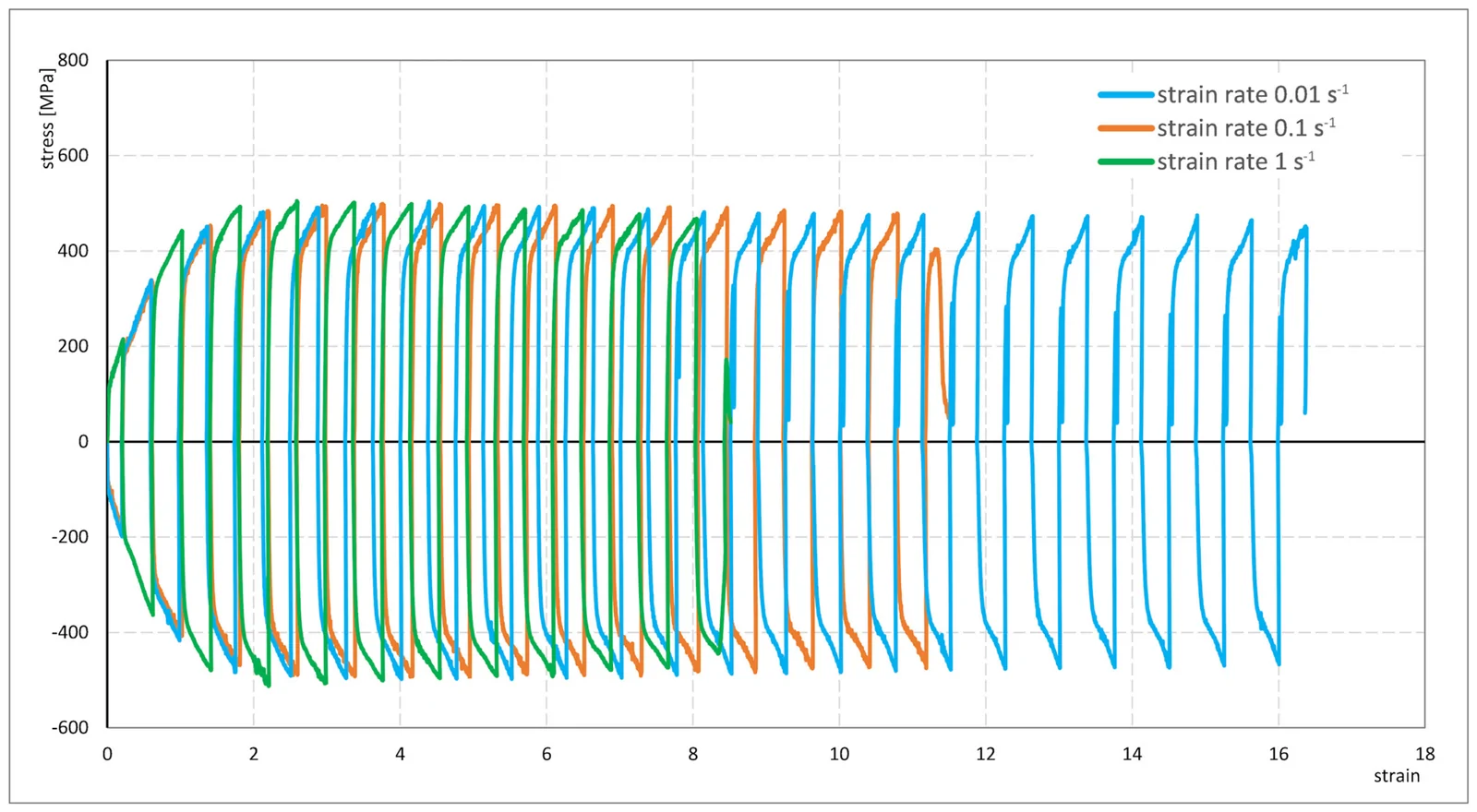
Comparison of cyclic torsion curves at a strain amplitude of 0.3 and all investigated strain rates.

All the above-presented cyclic torsion results demonstrate that for each of the applied strain rates, both the flow stress value markedly increases and the strain level decreases with an increasing strain amplitude, which is caused by the enhanced strain hardening of the material. To demonstrate the effect of the strain amplitude on the deformability of the CuZn30 brass at room temperature, Figure 17, Figure 18 and Figure 19 present a comparison of the stress–strain curves for all strain rates—0.01 s^−1^, 0.1 s^−1^ and 1 s^−1^—with strain amplitudes of 0.03, 0.06, and 0.3, respectively.

The curves presented below in Figure 17, Figure 18 and Figure 19 are consistent with the previous interpretation of the results obtained in the monotonic tests regarding the stress level. For the strain amplitude of 0.03, the results at the strain rate of 1 s^−1^ are characterized by the highest stress, whereas the lowest stress was obtained at the strain rate of 0.01 s^−1^. This stems from the fact that, although the CuZn30 brass is characterized by low strain rate sensitivity, the change in stress is nevertheless noticeable, particularly at smaller strains. This is clearly visible in the monotonic tests, e.g., for a strain of 0.25 in Figure 4.

When analysing the strain amplitudes for all the plots presented in Figure 17, Figure 18 and Figure 19, it can be observed that the strain rate has a significant effect on the limit strain, which is defined as the strain value at the moment of material failure. Decreasing the strain rate reduces the work hardening rate of the material and accelerates the restoration processes of the deformed microstructure. This generally leads to an increase in limit strains, as shown in Figure 17, Figure 18 and Figure 19. In the case of a higher strain rate, a larger number of cycles occurs per unit of time. This leads to faster hardening of the material and, consequently, to earlier failure of the specimen.

The results obtained in the conducted tests proved that by controlling the strain path, it is possible to significantly increase the strain and reduce the stress of the CuZn30 brass. At cold working temperatures, this stems primarily from the forced 180° change in the deformation direction, which leads to the generation of dislocations of opposite signs relative to the dislocations residing in the pile-ups formed during deformation in the primary direction. This creates conditions for their mutual annihilation. The motion direction of the piled-up dislocations also changes, taking place under a backstress—a lower stress originating from the piled-up. The aforementioned phenomena are described by the Bauschinger effect, which is addressed by the kinematic hardening hypothesis of the material; this hypothesis assumes that during the plastic forming process, the yield surface does not alter its shape and dimensions but shifts as a rigid body by a specific vector in the stress space.

## 6. Conclusions

As follows from the conducted research, the application of complex strain paths, including symmetrical cyclic deformation, significantly contributes to increasing the limit strains and altering the flow stress compared to monotonic deformation. The influence of the applied deformation methods on the flow stress is not unambiguous. It certainly depends on the temperature, strain rate, and material type, but above all, on the applied strain path. The positive effect of a forced strain path change on reducing the flow stress can be observed across the entire strain range only at low strain amplitude values, generally below 0.3. In general, it can be stated that large strain amplitudes exceeding 0.3 do not significantly affect the flow stress level in relation to the results obtained during the monotonic deformation of the investigated material. The obtained results allow for the assumption that by applying an appropriate strain path in industrial manufacturing processes, an improvement in deformability and a reduction in the flow stress of the CuZn30 brass can be expected.

In summary, the following conclusions can be drawn from the obtained results:

1. A correct interpretation of the research results when comparing stress–strain curves in cyclic and monotonic deformation processes requires the application of a physically equivalent description of strain. Such a strain is the plastic strain, rather than the total strain, in which the contribution of elastic strains in cyclic processes is entirely different from that in monotonic processes.
2. The strong strain hardening of the CuZn30 brass under cold deformation conditions is a direct consequence of the low stacking fault energy of this alloy, which restricts dynamic recovery. However, the introduction of oscillatory torsion (causing a 180° change in the strain path) stimulates dynamic recovery processes. By modifying the dislocation structure and generating conditions favorable for dislocation annihilation, this scheme allows for effective counteraction against the effects of strain hardening.
3. The application of a forced strain path change in a symmetrical cyclic torsion test enables the control of stress and strain. By altering the strain path, it is possible to significantly increase the limit strains and reduce the flow stress.
4. The application of oscillatory cyclic torsion with small strain amplitudes (0.03 and 0.06) allows a large increase in the limit strain to be achieved compared to the monotonic test. Applying an amplitude that is an order of magnitude larger (0.3) has a much smaller effect on the strain increase.
5. The high ductility in cyclic tests with small amplitudes (0.03 and 0.06) is accompanied by a significant reduction in plastic flow resistance. The maximum flow stress is reduced by up to 2.5 times (for the amplitude of 0.03) relative to the monotonic test and remains at a nearly constant level throughout the entire duration of the test. In the case of the 0.3 amplitude, the stress level is close to that obtained in the monotonic torsion test.
6. An increase in the strain rate during oscillatory torsion negatively affects the limit strain. The largest increase in deformability was obtained for the lowest strain rate (0.01 s^−1^), whereas for the highest strain rate (1 s^−1^), this increase was the smallest due to the larger number of cycles per unit of time and faster hardening of the material. For each of the investigated strain rates, an unambiguous relationship occurs: with an increasing cyclic strain amplitude, a distinct increase in the flow stress is accompanied by a concurrent, drastic drop in the limit strain (to failure).
7. Varying the strain rate has virtually no effect on changing the microstructure grain size during minor-cyclic torsion. Conversely, an increase in the strain amplitude enables effective refinement of the CuZn30 brass microstructure without the unidirectional grain elongation characteristic of monotonic torsion, which promotes the retention of more isotropic mechanical properties of the material while simultaneously achieving surface strengthening.
8. The research results unambiguously prove that by precisely controlling the parameters of cyclic deformation (the rate and amplitude), it is possible to flexibly tailor the flow stress and limit strain of the CuZn30 brass during forming at the cold working temperature.
9. The results of the presented research, in conjunction with modelling and computer-aided design, can be utilized to develop a proposal for optimizing the existing technological solutions for the plastic forming processes of the CuZn30 brass, as well as to establish potential foundations and guidelines for developing new, energy-efficient, and high-performance technologies for manufacturing products from the investigated material. However, this requires further research, which should include, among others, the behaviour of the investigated material during deformation with a forced strain path change at elevated temperatures.

## Figures and Tables

**Table 1:** Percentage chemical composition of tested CuZn30 brass [3].

Material	Cu	Zn	Fe	Ni	As	Mn
**CuZn30**	70.1	residue	0.01	0.001	0.001	0.004
	**Bi**	**Sn**	**Al**	**Si**	**Cd**	**Sb**
	0.001	0.03	0.001	0.001	0.001	0.001

**Table 2:** Cyclic torsion results at a strain rate of 0.01 s^−1^ and all investigated amplitudes.

Test Conditions	Stress [MPa]	Strain
$\dot{\varepsilon }$ = 0.01 s^−1^/*ε_ac_* = 0.03	231	164
$\dot{\varepsilon }$ = 0.01 s^−1^/*ε_ac_* = 0.06	287	138
$\dot{\varepsilon }$ = 0.01 s^−1^/*ε_ac_* = 0.3	495	16.4

**Table 3:** Cyclic torsion results at a strain rate of 0.1 s^−1^ and all investigated amplitudes.

Test Conditions	Stress [MPa]	Strain
$\dot{\varepsilon }$ = 0.1 s^−1^/*ε_ac_* = 0.03	260	108
$\dot{\varepsilon }$ = 0.1 s^−1^/*ε_ac_* = 0.06	315	78
$\dot{\varepsilon }$ = 0.1 s^−1^/*ε_ac_* = 0.3	500	11

**Table 4:** Cyclic torsion results at a strain rate of 1 s^−1^ and all investigated amplitudes.

Test Conditions	Stress [MPa]	Strain
$\dot{\varepsilon }$ = 1 s^−1^/*ε_ac_* = 0.03	276	75
$\dot{\varepsilon }$ = 1 s^−1^/*ε_ac_* = 0.06	308	55
$\dot{\varepsilon }$ = 1 s^−1^/*ε_ac_* = 0.3	509	8.5

## Data Availability

The original contributions presented in this study are included in the article. Further inquiries can be directed to the corresponding author.
